# *β*-Ketoallylic methylsulfones synthesis via inert C(*sp*^3^)–H bond activation by magnetic Ag–Cu MOF

**DOI:** 10.1038/s41598-023-49670-5

**Published:** 2023-12-18

**Authors:** Firouz Matloubi Moghaddam, Atefeh Jarahiyan, Parisa Yaqubnezhad Pazoki

**Affiliations:** Laboratory of Organic Synthesis and Natural Products, Department of Chemistry, Sharif University of Technology, Azadi Street, PO Box 111559516, Tehran, Iran

**Keywords:** Organic chemistry, Chemistry

## Abstract

Herein, the one-pot tandem synthesis of *β*-ketoallylic methylsulfones has been achieved from readily available dimethyl sulfoxide and acetophenones as coupling partners in one step. In this procedure, dimethyl sulfoxide serves as a triple role including solvent, dual synthon and as an oxidant agent. The use of magnetic Ag–Cu MOF as a bimetallic catalyst is the key to the progress of the reaction due to its accessible active sites. It provides facile access to various *β*-ketoallylic methylsulfone derivatives from direct C(*sp*^3^)–H bond activation and functionalization of aromatic methyl ketones especially acetophenones with electron-rich and electron-poor groups. Moreover, the present work offers a synthetically powerful strategy to form products in good to excellent yields (74–96%) with the atom, step, and pot economics. It has also delivered a new chromane-4-one derivative from 2-hydroxy acetophenone with intramolecular Michael-addition of related *β*-ketoallyl methylsulfone product. In the final step, the electronic properties of some products have been predicted with the theoretical studies.

## Introduction

The sulfonyl functional group (–SO_2_–) has played a significant role in the development of efficient methods in organic synthesis. Molecules containing sulfone group are a main class of compounds due to their broad utility in natural products, agrochemicals, and organic chemistry^1^. Moreover, Sulfones as common sulfur-containing reagents constitute a wide range of biological active scaffolds and drug molecules^2^. The use of sulfone as a coupling partner in cross-coupling reactions is also a powerful tool to access complex molecules^3^. In this regard, they can give tremendous functionality toward the activation of adjacent positions in versatile approaches in which the reaction continues with desulfonation^4^.

Given the important role of dimethyl sulfoxide (DMSO) in some organic reactions, it has recently received great attention. DMSO has been typically used as a less toxic and inexpensive solvent with high polarity to dissolve polar and nonpolar compounds^5^. Up to now, limited reports have been introduced DMSO as a mono- and dual-synthons source C, SCH_3_, SO_2_CH_3_, CH, CH_3_, CH_2_, CHO, and O to synthesize worthwhile building blocks.

A number of methods have been developed to activate DMSO serving as various reagents^5–11^. However, DMSO activation toward use as a dual-synthon will be challenging for chemists. Thiomethylated flavones and furans have been achieved by Guo’s group in the presence of I_2_, K_2_S_2_O_8,_ and DMSO as a dual-synthon^12^. Wei and co-workers developed the synthesis of Co(III)-catalyzed *β*-amino ketones from amide, ketone, and DMSO^13^. Recently, Rode et al. obtained the *β*-acyl allyl sulfones derivatives of some acetophenones/aryl acetylene with selectfluor and DMSO acting as dual-synthon^14^.

There are limited reports on the synthesis of *β*-acyl allyl sulfones suffering from the presence of silver salt, transition metals, and phase transfer catalysts^14^. For example, the Fe-catalyzed synthesis of *β*-acyl allyl sulfones has been described by Deng and co-workers^15^. This multi-component reaction has successfully proceeded with sodium sulfonate, acetophenone, sodium dodecyl sulfate, and K_2_S_2_O_8_ oxidant. To overcome disadvantages, development of efficient and applicable strategies is highly desirable for *β*-acyl allyl sulfones synthesis.

C–H bond activation strategies have been well documented for the construction of carbon–carbon bonds and there are numerous number reports of C(*sp*^2^)–H activation but inert C(*sp*^3^)–H bond faces more challenges for activation owing to high bond dissociation energy. In a comparison with different transition metals, palladium-catalyzed approaches have been more applied to active C(*sp*^3^)–H due to its considerable reactivity^16^. Therefore, focusing on readily available metals instead of precious palladium will be worthy for carbon–carbon formation via C(*sp*^3^)–H bond activation.

**Figure 1:**
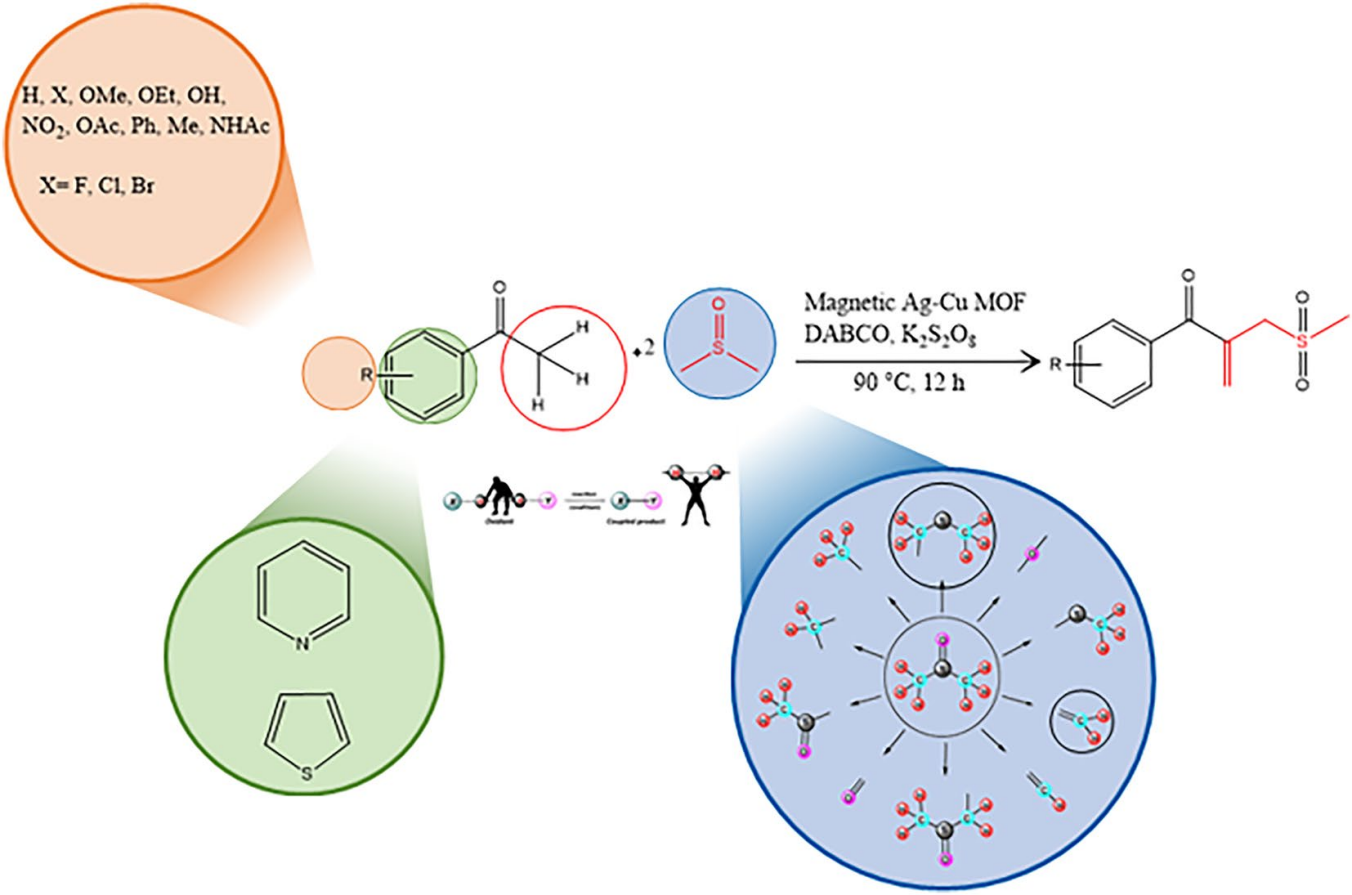
Synthesis of *β*-ketoallyl methylsulfones via solvent activation strategies.

In the present work, the *β*-ketoallyl methylsulfones synthesis is described through the DMSO activation and C(*sp*^3^)–H activation of acetophenones with earth-abundant metals. To the best of our insight, this is the first report to access *β*-ketoallyl methylsulfones in the presence of magnetic Ag–Cu MOF catalyst. In this protocol, DMSO plays a triple role including solvent, dual synthon, and oxidant to form two C–C and one O=S=O bonds (Fig. 1).

## Experimental section

### General remarks

All chemical materials and solvents were commercially purchased and used without further purification. Analytical thin layer chromatography (TLC) was performed on Merck TLC silica gel plates. A Bruker Avance DRX-500 (125 MHz) spectrometer at ambient temperature was used to record ^1^H-NMR and ^13^C-NMR spectra. An ABB Bomem MB-100 FTIR spectrometer was used to obtain Fourier transform infrared plots in the 4000–400 cm^−1^ range. The X-ray diffraction data were collected on an analytical diffractometer with a copper target at 40 kV and 40 mA and Cu Kα (λ = 1.54 Å) for 2*θ* in the range of 0°–85°. The composite morphology was obtained from Philips CM 120, 100 kV with transmission electron microscopy (TEM) and TESCAN–Mira III at 15 kV for field emission scanning electron microscopy (FE-SEM). The measurement of porosity and specific surface area of the catalyst was investigated with Belsorp- mini II device. Determining the concentration of elements was done with Varian (730-ES). Agilent Technologies (5975C) devise was used for mass analysis of organic compounds.

### Synthesis of catalyst

#### Preparation of magnetic polyacrylic acid (Fe_3_O_4_@PAA)

The Fe_3_O_4_@PAA as a magnetic substrate was synthesized in a stepwise procedure according to our previous work^17^.

#### Preparation of magnetic Ag–Cu MOF composite

Magnetic Ag–Cu MOF composite was prepared with a solvothermal method as follows^18^: 2.4 g (10 mmol) of Cu(NO_3_)_2_·3H_2_O and 1.7 g (10 mmol) of AgNO_3_ were dissolved homogeneously in 150 mL of dimethylformamide (DMF) at room temperature under magnetic stirring. Then, 1.0 g of Fe_3_O_4_@PAA was mixed into the prepared solution. Thereafter, a mixture of linkers containing solution of terephthalic acid (H_2_BDC, 20 mmol, 3.3 g) as the main linker and benzoic acid (BzOH, 1 mmol, 122 mg) as the defective linker in 150 mL of DMF was added to the above solution at room temperature. After stirring for 1 h, the mixture was transferred into an autoclave and heated at 120 °C for 24 h. DMF solvent of the formed mixture was replaced with chloroform while stirring for 24 h. The black–green colored magnetic Ag–Cu MOF precipitate was separated with an external magnet and dried in an oven at 60 °C for 24 h.

### General procedures for magnetic Ag–Cu MOF mediated synthesis of *β*-ketoallyl sulfones

In a test tube, acetophenone (0.2 mmol), DABCO (0.1 mmol) and magnetic Ag–Cu MOF (5.0 mg) were mixed in DMSO (1.2 mL) and stirred at room temperature for 5 min. Thereafter, K_2_S_2_O_8_ (0.4 mmol) were added and the mixture was stirred at 90 °C in an oil bath for 12 h under atmospheric conditions. The reaction was monitored by TLC (ethyl acetate: *n*-hexane, 4: 5), and after reaction completion, the dark brown resulting solution was cooled to room temperature. Next, the catalyst was precipitated and separated from the solution by an external magnet. Then, the solution was diluted by ethyl acetate and water, dried over anhydrous Na_2_SO_4_ and concentrated in a vacuum. Finally, the yellow oil product was purified by column chromatography, and confirmed by ^1^H-NMR and ^13^C-NMR.

### Acid–base back titration

The titration experiment was done as follows: 100 mL of standard sodium hydroxide solution (0.00848 M) was placed into a round bottom flask containing 250 mg of magnetic Ag–Cu MOF and stirred for 24 h^19^. Thereafter, pH was equal to 6; thus, 3.5 mL of the denser standard sodium hydroxide solution (0.0848 M) was added and stirred for 4 h until pH reached about 11. In the next step, the catalyst was filtered off and washed with the deionized water. Finally, the colorless filtrate was back-titrated by 52.6 mL of standard hydrochloric acid (0.001 M) in the presence of phenolphthalein indicator (2 drops). Notably, standard sodium hydroxide solutions were prepared by 0.001 M of standard hydrochloric acid solution.

Calculations:

- Initial amount of sodium hydroxide: (100 mL × 0.00848 M) + (3.5 mL × 0.0848 M) = 1.1448 mmol
- Final amount of sodium hydroxide: hydrochloric acid consumption amount = 52.6 mL × 0.001 M = 0.0526 mmol
- Consumption amount of sodium hydroxide: 1.1448 mmol − 0.0526 mmol = 1.0922 mmol
- Acidity of the prepared catalyst: (1.0922 mmol/0.25 g): 4.3688 mmol/g

## Results and discussion

MOFs as porous coordination networks are constructed from rigid organic linkers and inorganic nodes coordination bonds. The organic likers, which generally include carboxylic acid groups, can show acidic properties. On the other hand, metals that form nodes play an important role as Lewis acids. Also, the acidic character of the composite can be improved by creating defects in the structure. In this work, defects are created by using 5 mol% of benzoic acid as a modulator linker. As a result, the functional groups of starting materials will be activated better by increasing the acidic character of the catalyst. Therefore, the reaction will proceed with high efficiency in the presence of the catalyst with its acidic nature.

**Figure 2:**
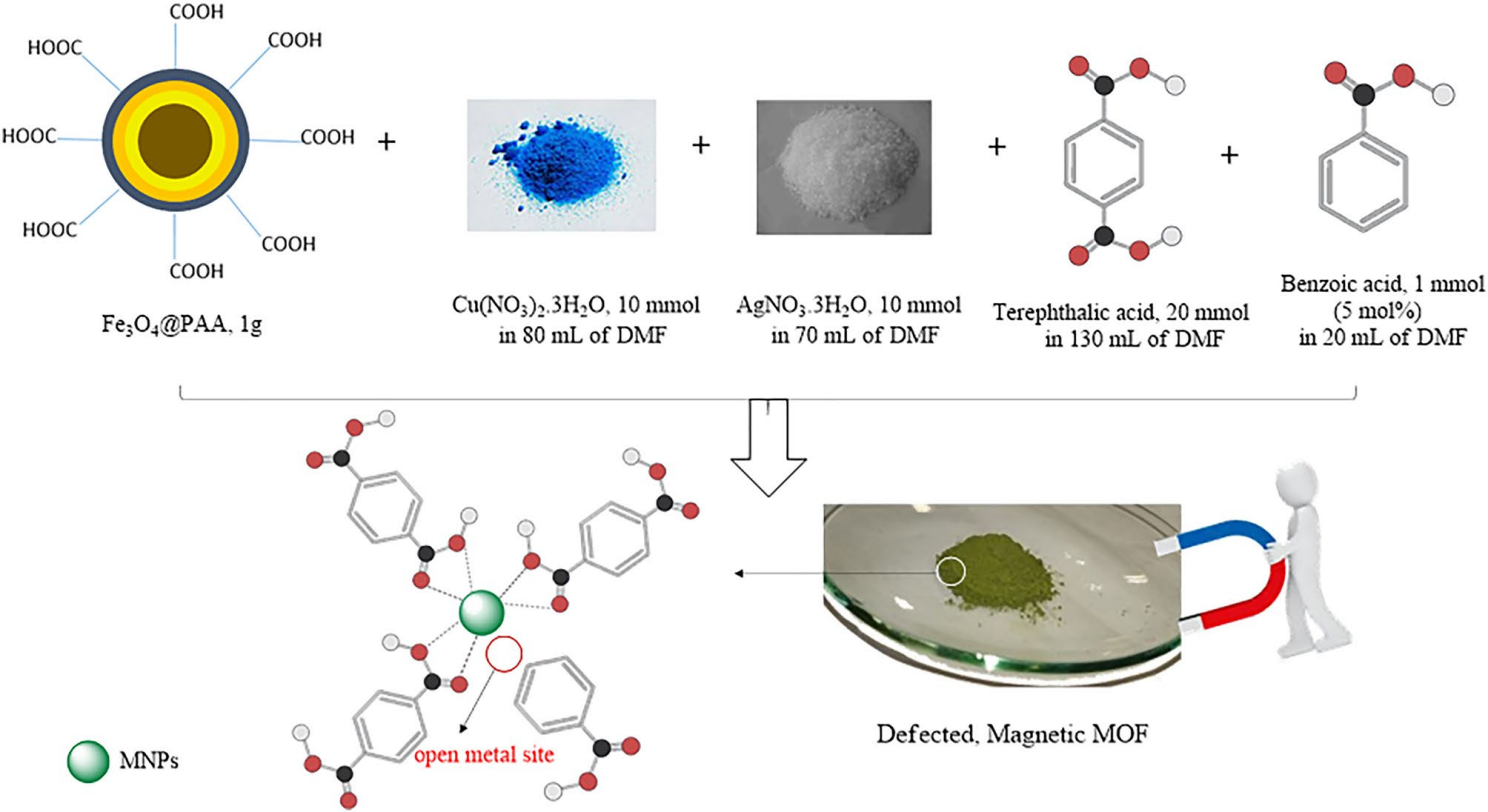
Preparation of magnetic Ag–Cu MOF composite.

Having application of MOF in the field of catalyst^20^, magnetic Ag–Cu MOF catalyst was prepared through a stepwise protocol as presented in Fig. 2 and then a series of characterization was performed to confirm catalyst structure. After that, a model substrate including 4-methoxy acetophenone (**1a**), DMSO as a reagent, solvent, and oxidant (**2**), and magnetic Ag–Cu MOF catalyst was carried out to the synthesis of *β*-ketoallyl methylsulfones in good to high yields.

### Catalyst characterization

#### FT-IR spectroscopy

**Figure 3:**
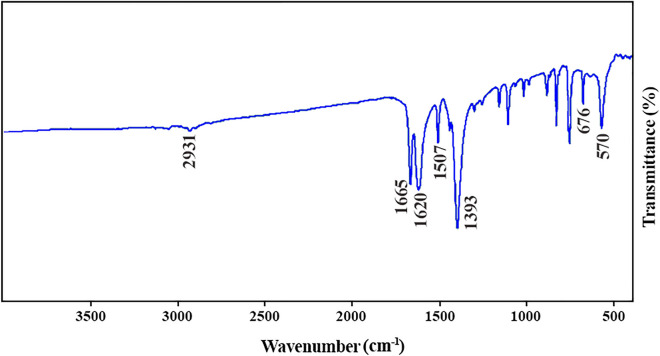
FT-IR spectrum of magnetic Ag–Cu MOF.

Figure 3 displays the FT-IR spectrum of magnetic Ag–Cu MOF catalyst in the range of 4000–400 cm^−1^ to confirm the presence of catalyst functional groups. The characteristic absorption bands in the range of 1200–1000 cm^−1^ and 1000–700 cm^−1^ are related to the in-plane and out-of-plane C–H bending modes of the linkers, respectively. The band at 2931 cm^−1^ is ascribed to the stretching vibration of C–H groups of linkers^18,21^. The presence of C–O groups of linkers is proved by the characteristic band at 1393 and 1620 cm^−1^. In addition, two peaks at 570 and 676 cm^−1^ relate to metal–oxygen stretching vibrations^18,22^. Moreover, bands at 1507 and 1665 cm^−1^ are owing to C–O groups of free carboxylic acids.

#### XRD analysis

**Figure 4:**
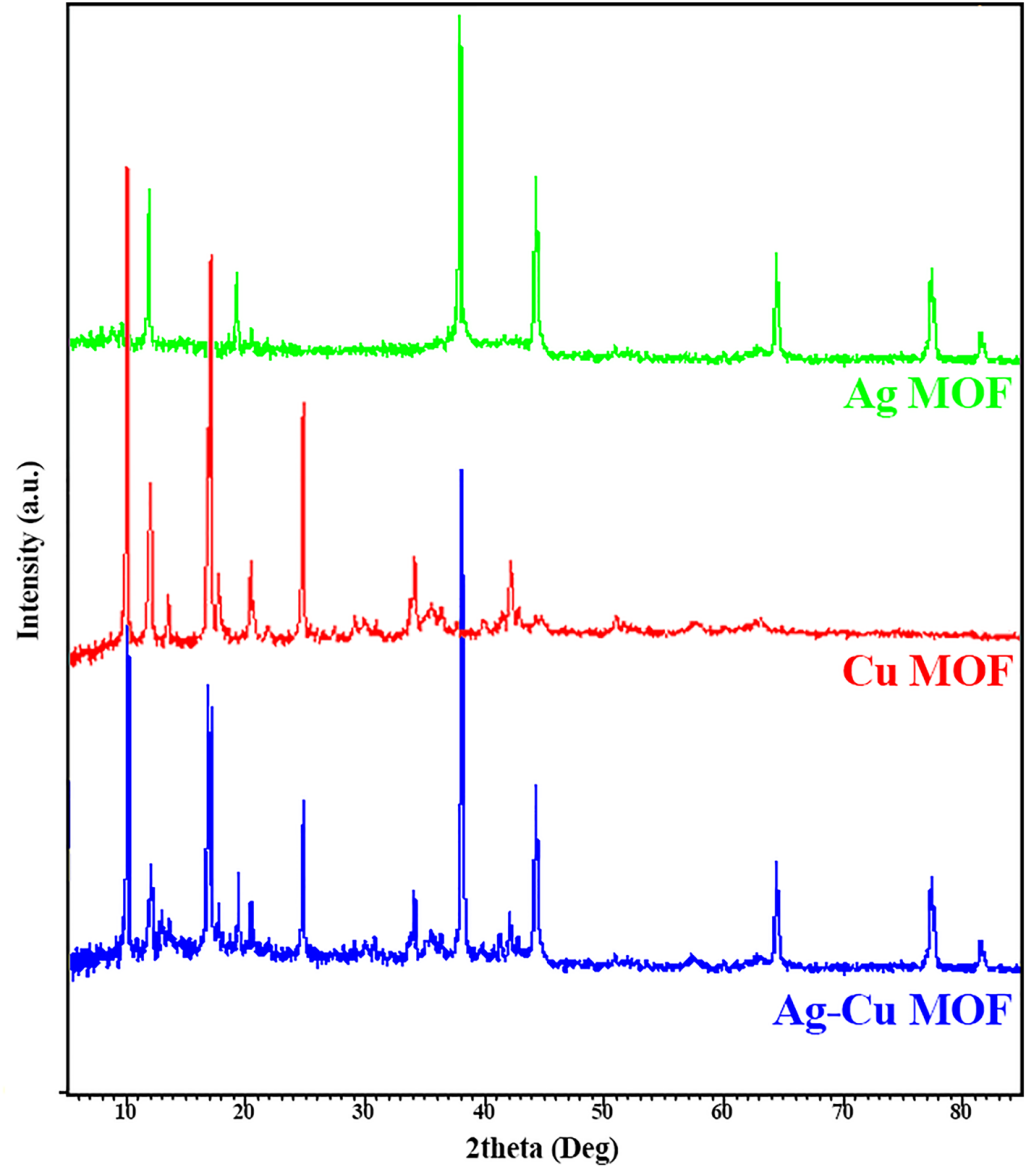
XRD patterns of magnetic Ag MOF, magnetic Cu MOF, and magnetic Ag–Cu MOF.

A crystalline structure description of magnetic Ag–Cu MOF was gained by its XRD pattern (Fig. 4). The diffraction peaks at 2*θ* = 10.2°, 12.1°, 16.8°, 17.2°, 17.7°, 19.4°, 20.4°, 24.8°, 34.1°, 38.1°, 42.1°, 44.2°, 64.4°, 77.3°, and 81.5° are related to magnetic Ag–Cu MOF structure. For a more detailed explanation, the peaks at 2*θ* = 12.0°, 16.8°, 38.1°, 44.2°, 64.4°, 77.3°, 81.5° and peaks at 10.2°, 12.1°, 17.2°, 20.4°, 24.8°, 34.1°, 42.1° match with Ag-MOF and Cu-MOF, respectively^18^. Therefore, the successful synthesis of the bimetallic MOF is confirmed. It is worth noting, the magnetic nature of the prepared MOF has been observed experimentally through separation by an external magnetic field and also the presence of Fe and Si elements in the EDX analysis.

#### Morphology study

**Figure 5:**
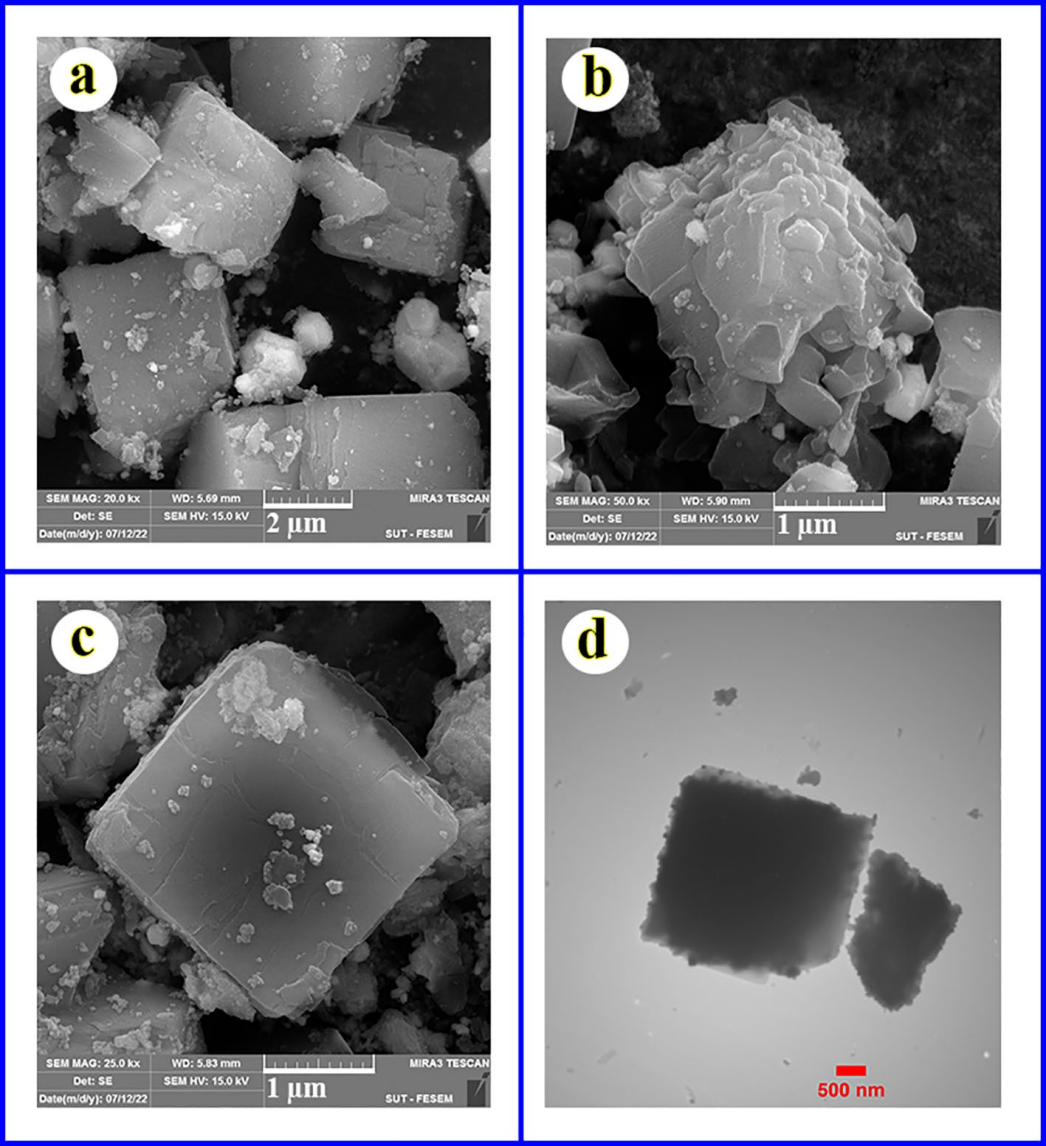
FE-SEM (**a**–**c**) and TEM (**d**) of magnetic Ag–Cu MOF.

The FE-SEM and TEM images of magnetic Ag–Cu MOF are presented in Fig. 5. The FE-SEM images clearly reveal the cubic structure of the frameworks (Fig. 5a–c). In addition, some frameworks show porous and layered morphology (Fig. 5b). The TEM image (Fig. 5d) also confirms the porous and cubic structure of magnetic Ag–Cu MOF.

#### EDX analysis and elemental mapping

**Figure 6:**
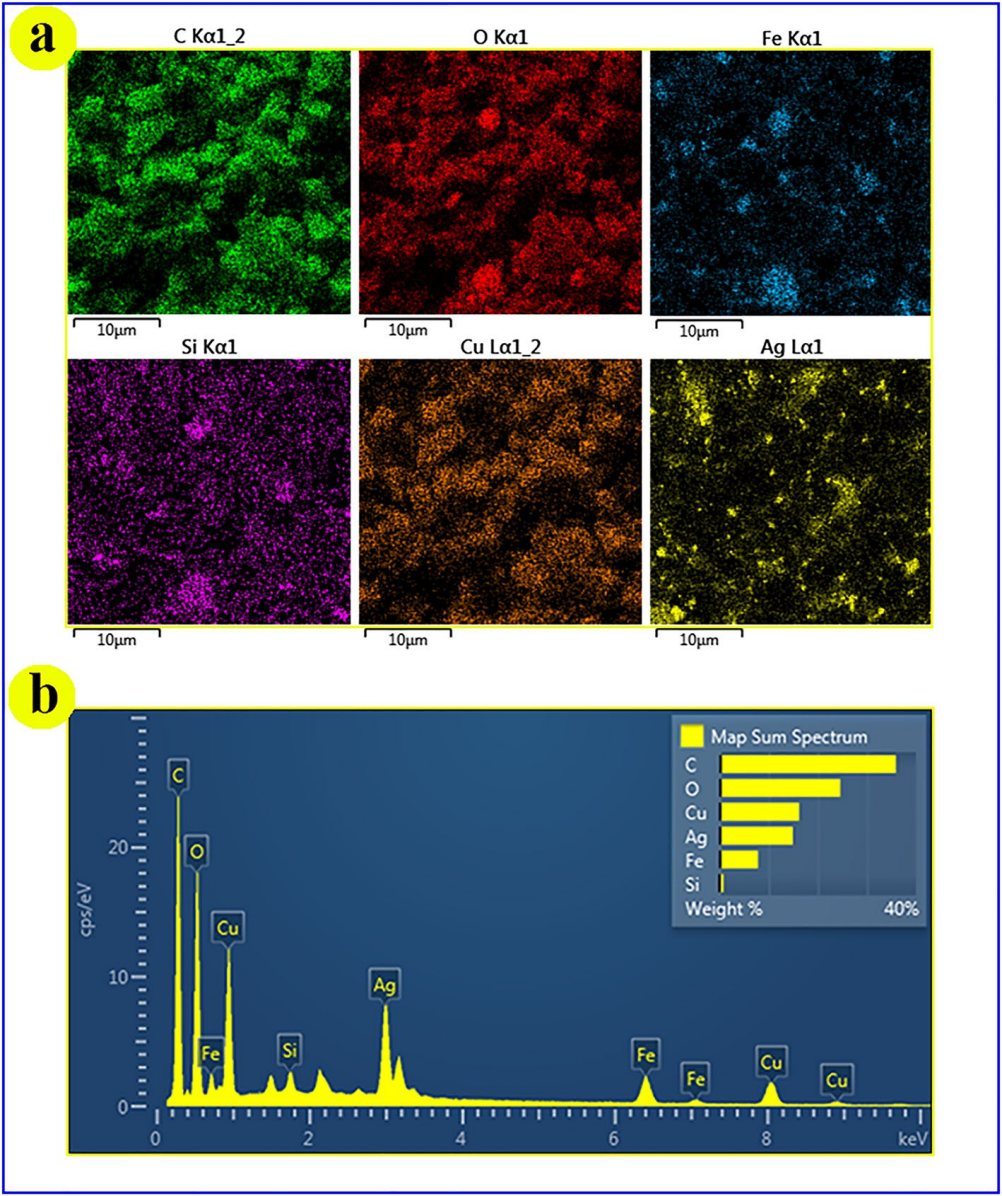
(**a**) Elemental mapping and (**b**) EDX analysis of magnetic Ag–Cu MOF.

The composition of the prepared MOF including Cu, Ag, C, Fe, and Si is confirmed by EDX analysis (Fig. 6b). The elemental mapping of Cu and C shows clearly their cubic structure while the elemental mapping of Ag reveals its dispersion. Then, it can be concluded that Ag elements are doped into the main frameworks (Fig. 6a).

#### Brunauer–Emmett–Teller (BET) surface area analysis

The nitrogen adsorption–desorption data analysis proves the porosity of the fabricated magnetic Ag–Cu MOF. The surface area value of 1054.61 (m^2^/g) was obtained for magnetic Ag–Cu MOF. The BET amount comparison between magnetic Ag–Cu MOF as a bimetallic MOF and Cu-MOF (474.68 (m^2^/g)) as a monometallic one confirms the bimetallic nature of magnetic Ag–Cu MOF based on the pervious reported article^18^.

#### Vibrating sample magnetometer (VSM) analysis

**Figure 7:**
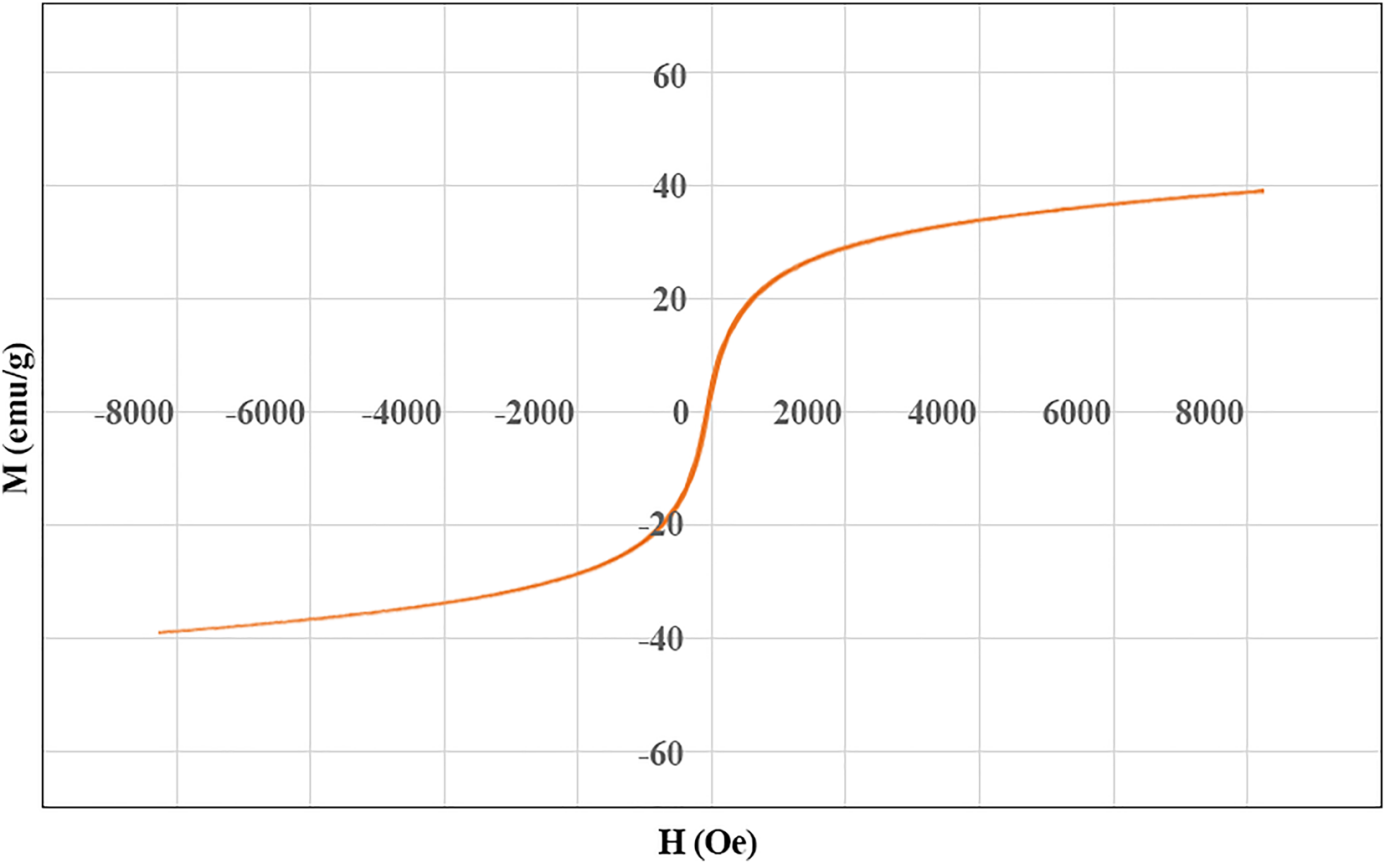
VSM curve of magnetic Ag–Cu MOF.

The magnetization curve of magnetic Ag–Cu MOF at room temperature is presented in Fig. 7. The saturation magnetization of the composite shows its superparamagnetic properties. It helps to separate the sample with an external magnet and its recyclability.

#### Acid–base back titration

To determine the surface acidity of magnetic Ag–Cu MOF, the back titration was used based on the reported literature^19^. Analysis of the titration was done and found that one gram of the catalyst is equal to 4.3688 mmol of H^+^ cation in water. It means the prepared catalyst has highly acidic properties.

#### ICP-OES analysis

Inductively Coupled Plasma Optical Emission spectroscopy was done to investigate the concentration of Cu and Ag elements of magnetic Ag–Cu MOF and results are summarized in Table 1. In detail, sample 1 is related to 200 mg of magnetic Ag–Cu MOF, and sample 2 is related to 200 mg of magnetic Ag–Cu MOF after being placed in the reaction conditions (60 ml of dimethyl sulfoxide solvent, at 90 °C for 12 h). The percentage of Cu element of samples 1 and 2 is 12.2 and 7, respectively and Ag element is 18.7 and 9.1, respectively. According to the data, Cu is almost preserved in the metal–organic frameworks while small amount of silver is released confirming EDX results.

**Table 1:** Data of ICP-OES analysis.

Sample labels	Element	Wavelength	Element	Wavelength
	Ag	327.068	Cu	327.395
Blank (%)	0.0		0.0	
1 (%)	18.7		12.2	
2 (%)	9.1		7.0	

### C(***sp***^3^)–H bonds activation for ***β***-keto allyl methylsulfones synthesis over magnetic Ag–Cu MOF catalyst

At the outset of this project, we commenced our study with 4-methoxyacetophenone (**1**), dimethyl sulfoxide (**2**) summarized in Table 2, and *β*-ketoallyl methylsulfone product (**3**) was obtained in the highest yield (96%) with magnetic Ag–Cu MOF in the presence of 1,4-diazabicyclo[2.2.2]octane (DABCO) and K_2_S_2_O_8_ at 90 °C for 12 h (Table 2, entry 1). Other bases such as 1,8-diazabicyclo [5.4.0] undec-7-ene (DBU), 1-methyl imidazole (NMIz), 4-dimethylaminopyridine (DMAP), *N*,*N*-dimethylaniline (DMA), tetramethylethylendiamine (TMEDA) and *N*,*N*-dimethyl-*p*-toluidine (DMPT) were investigated instead of DABCO but the desired product was obtained in the low yields (Table 2, entries 2–8). Only a trace amount of the desired product was achieved with inorganic bases such as K_2_CO_3_, KHCO_3,_ and NaOAc (Table 2, entries 9–11). Since the reaction was sensitive to additives, we employed others such as Na_2_S_2_O_8_, TBHP, H_2_O_2,_ and (BzO)_2_. A trace amount of the product was detected for Na_2_S_2_O_8_ and no reaction proceeded with other additives (Table 2, entries 12–15). Moreover, no reaction occurred in a control experiment without base or additive under the optimal conditions (Table 2, entries 16–18). In addition, it was found that a trace amount of the product (**3**) was formed in the presence of nitrogen gas (Table 2, entry 19). The use of lower amount of the catalyst resulted in a lower yield (Table 2, entry 20). Other catalysts such as Cu(NO_3_)_2_ and monometallic frameworks (Ag-MOF and or Cu-MOF) instead of the prepared catalyst exhibited low reactivities (Table 2, entries 21–23). Worth mentioning, Cu-MOF afforded just *α, β*-unsaturated product (intermediate D in mechanism). A brief examination of temperature showed the reaction proceeded to 96% at 90 °C (Table 2, entries 24–28). As a final optimization test, the desired product could not be obtained without the catalyst and any by-product was not detected (Table 2, entry 29).

**Table 2:** Optimization condition for C(sp^3^)-H activation reaction.


Entry	Base	Additive	Temp (℃)	Yield (%)
**1**	**DABCO**	**K**_**2**_**S**_**2**_**O**_**8**_	**90**	**96**
2	DBU	K_2_S_2_O_8_	90	18
3	Et_3_N	K_2_S_2_O_8_	90	15
4	NMIz	K_2_S_2_O_8_	90	10
5	DMAP	K_2_S_2_O_8_	90	23
6	DMA	K_2_S_2_O_8_	90	13
7	TMEDA	K_2_S_2_O_8_	90	25
8	DMPT	K_2_S_2_O_8_	90	28
9	K_2_CO_3_	K_2_S_2_O_8_	90	Trace
10	KHCO_3_	K_2_S_2_O_8_	90	Trace
11	NaOAC	K_2_S_2_O_8_	90	Trace
12	DABCO	Na_2_S_2_O_8_	90	Trace
13	DABCO	TBHP	90	–
14	DABCO	H_2_0_2_	90	–
15	DABCO	(BzO)_2_	90	–
16	DABCO	K_2_S_2_O_8_	90	Trace
17	–	K_2_S_2_O_8_	90	Trace
18	DABCO	–	90	Trace
19^a^	DABCO	K_2_S_2_O_8_	90	Trace
20^b^	DABCO	K_2_S_2_O_8_	90	74
21^c^	DABCO	K_2_S_2_O_8_	90	45
22^d^	DABCO	K_2_S_2_O_8_	90	–
23^e^	DABCO	K_2_S_2_O_8_	90	51
24	DABCO	K_2_S_2_O_8_	25	–
25	DABCO	K_2_S_2_O_8_	50	Trace
26	DABCO	K_2_S_2_O_8_	70	57
27	DABCO	K_2_S_2_O_8_	80	76
28	DABCO	K_2_S_2_O_8_	120	96
29^f^	DABCO	K_2_S_2_O_8_	90	–

4-Methoxyacetophenone (0.2 mmol), solvent (1.2 mL), base (0.1 mmol), MOF based-catalyst (5 mg), additive (0.4 mmol).

Significant values are in [bold].

^a^N_2_ atmosphere.

^b^Catalyst (2.5 mg).

^c^Ag MOF catalyst.

^d^Cu(NO_3_)_2_ catalyst.

^e^Ag-MOF and Cu-MOF.

^f^Without catalyst.

#### Substrate scope

With the optimal conditions in hand, we surveyed the generality of the C(*sp*^3^)–H activation reaction for the synthesis of *β*-ketoallylic methylsulfone products (**3**). As shown in Table 3, electron-rich and electron-deficient substrates were tested to gain different sulfonated *α, β*-unsaturated products (**3b, 3c**). The substituents at different positions (*ortho*-, *meta*-, *para*-) on the acetophenone gave the related *β*-ketoallylic methylsulfone products in good to excellent yields (**3b–3i, 3l–3o**). Acetophenones bearing alkyl, aryl, alkoxy, and phenyl groups provided desired products in high yields (**3i, 3l, 3b, 3n, 3m, 3h**). Acetophenones containing halogen groups served well in this reaction (**3d–3g**). The acetophenone derivative with π-extended could react well and provided product (**3i**) in good yield. It is worth noting that heteroaryl ketones such as pyridine and thiophene worked well and gave the *β*-ketoallylic methylsulfone products in good to excellent yields (**3j, 3k**). Non-aromatic methyl ketones could not proceed with this reaction (**3v, 3w**). Notably, the free hydroxyl group (**3s**) was compatible with this reaction (in the harsher than optimal condition, 120 °C for 24 h). The 2-acethyl benzoic acid derivative could not proceed with the current reaction. It may be related to intramolecular H-bonding of the carboxylic acid group and also coordination of groups containing oxygen to the open-metal sites of the catalyst (**3p**). 2-acethyl and 4-acethyl phenyl acetate derivatives served good selectivity in this reaction (**3q, 3r**). The protected amino derivative was tested due to the 4-amino acetophenone derivative (**3t**) could not deliver the desired product, and the product (**3u**) was obtained in excellent yield.

**Table 3:**
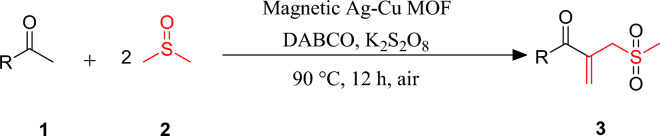
Substrate scope for the synthesis of β-ketoallylic methylsulfones.

Reaction conditions: acetophenones (0.2 mmol), DABCO (0.1 mmol), catalyst (5 mg), solvent (1.2 mL), K_2_S_2_0_8_ (0.4 mmol).

We were pleased to find that 2-hydroxy acetophenone derivative chromane-4-one product afforded in 120 °C for 24 h (Table 4, **3x**). It occurred with intramolecular Michael-addition of related *β*-ketoallyl methylsulfone product. Actually, the worthwhile methyl sulfone chromane-4-one was directly synthesized in one pot via coupling of the methyl group in the methyl ketone and DMSO.

**Table 4:**
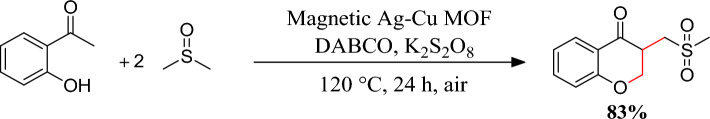
One-pot synthesis of methyl sulfone chroman-4-ones via coupling of methyl group in 2-hydroxy acetophenone and DMSO.

2-Hydroxy acetophenone (0.5 mmol), DABCO (0.25 mmol), catalyst (12.5 mg), DMSO (3 mL), K_2_S_2_O_8_ (1 mmol).

#### Mechanism pathway

**Figure 8:**
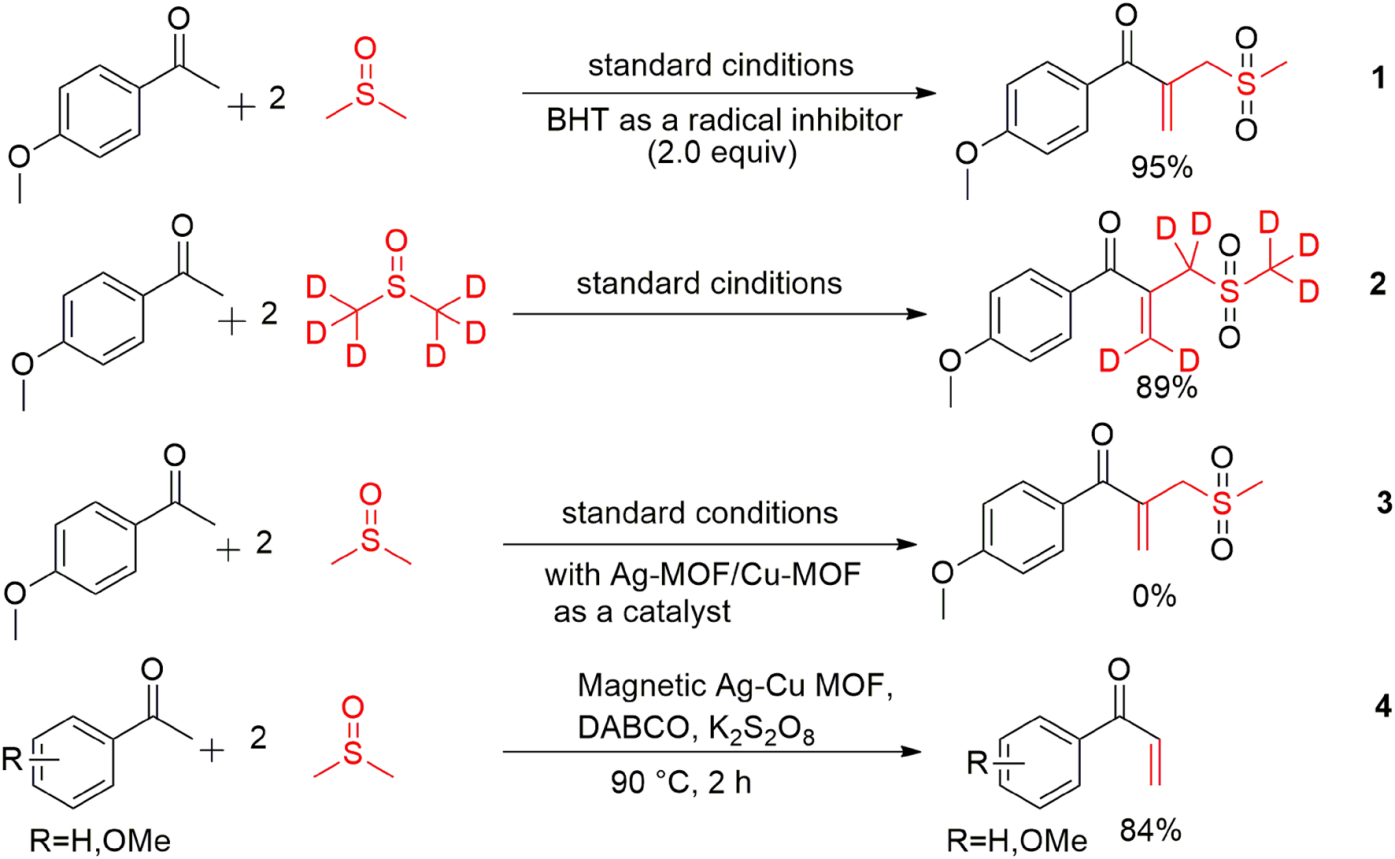
Control experiments.

To access insight into the mechanism of this C(*sp*^3^)–H activation reaction, several tests were performed (Fig. 8). Initially, the desired product was still afforded in high yield under the standard reaction conditions by addition BHT (2 equiv.) as a radical scavenger. Thus, it was concluded this reaction may not proceed by a radical process (Eq. 1). The reaction of 4-methoxy acetophenone with DMSO-*d*_*6*_ produced the deuterated product in 89% yield which was suggested that methylene and thioether groups come from DMSO (Eq. 2). It was found DMSO plays a triple role including solvent, oxidant and reagent, that it is a source for two functional groups. Worth mentioning, this reaction was investigated by monometallic Ag-MOF and Cu-MOF instead of the main catalyst and no favorite product was produced in both tests (Eq. 3). Furthermore, the *α, β*-unsaturated product was separated as a key intermediate under the standard reaction conditions after 2 h (Eq. 4).

**Figure 9:**
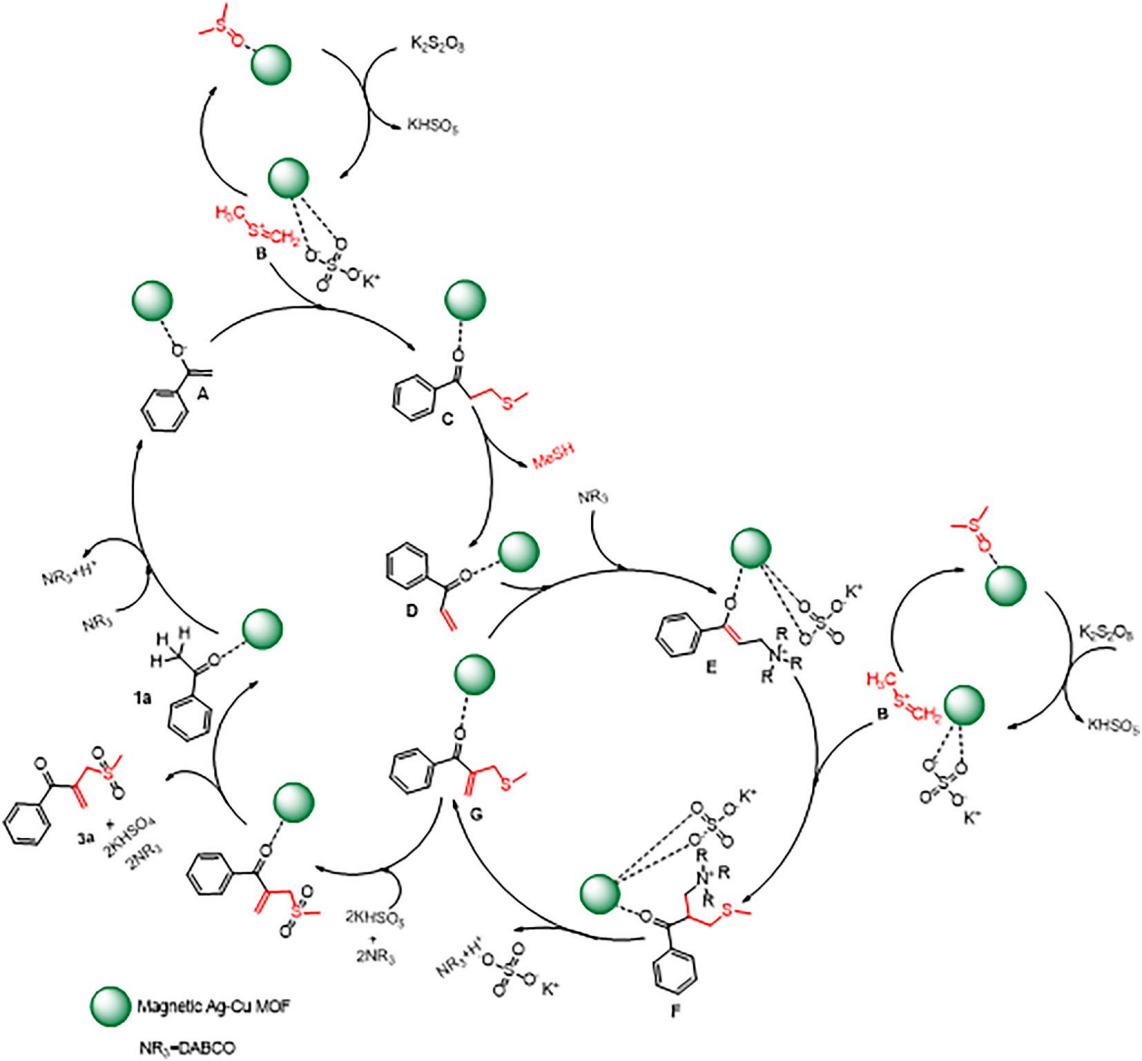
Proposed mechanism to synthesis of *β-*ketoallylic methylsulfone product.

**Figure 10:**
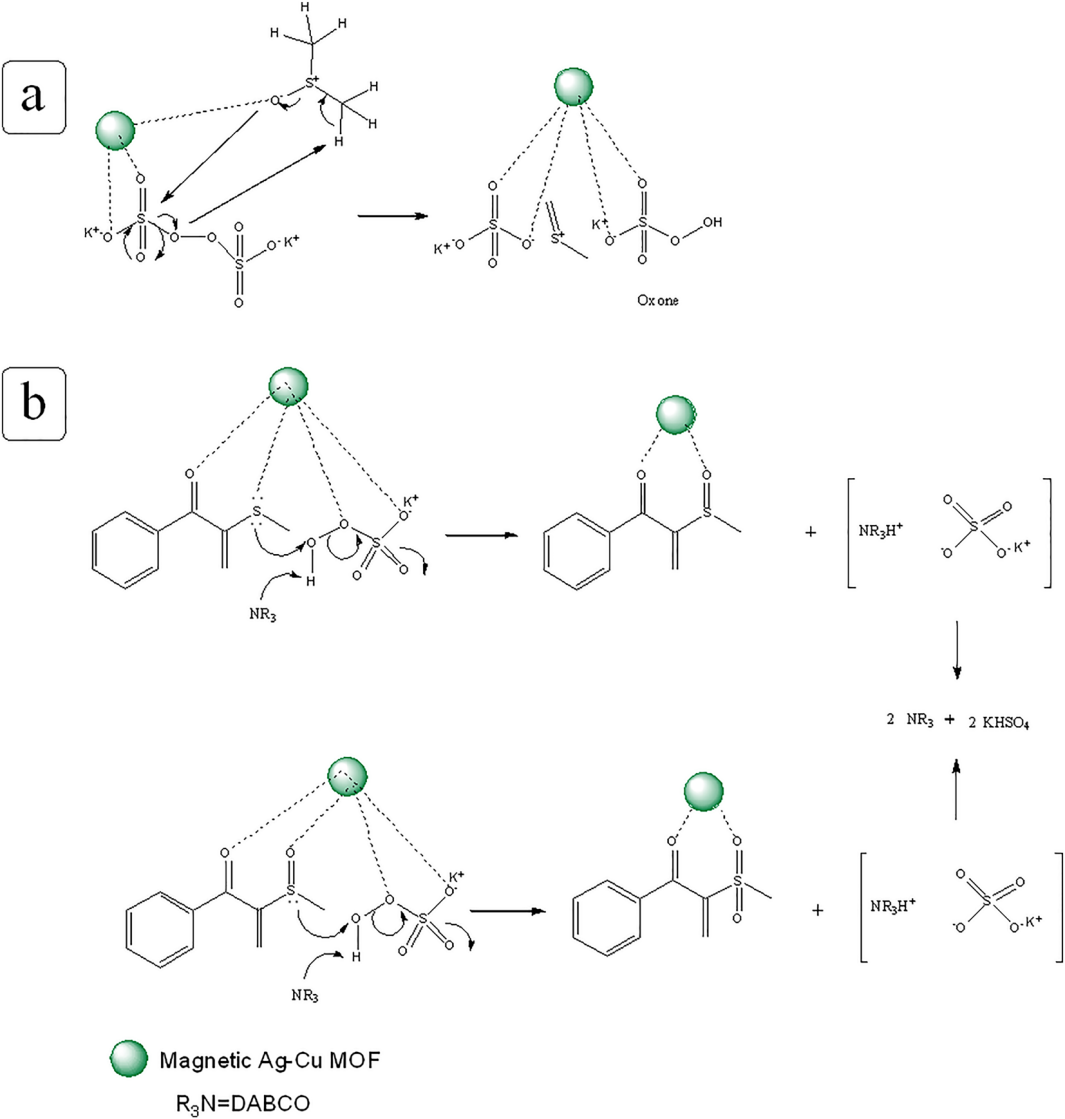
Proposed reaction path for generation of **B** species (**a**) and oxidation of **G** species (**b**).

Based on these observations and pervious articles^23^, a possible reaction mechanism is proposed for the C(*sp*^3^)–H activation reaction as shown in Fig. 9. Initially, DMSO is activated by K_2_S_2_O_8_ in the presence of the catalyst to give reactive electrophilic thionium ion (**B**), the mechanism of this step is proposed in details in Fig. 10a. Then, the intermediate C is generated from coupling of **A** and **B** (producing from **1a**). Thereafter, the intermediate **C** undergoes demethylthioation in the presence of K_2_S_2_O_8_ to generate **D** species and methanethiol. It is followed by Michael-addition with DABCO generating **E**, and then **E** undergoes electrophilic addition by **B** to provide **F** species. In next step, **G** species is formed with elimination of an ammonium salt from **F**. Finally, the desired product is generated from chemoselective oxidation of sulfide group of **G** by Oxone in the presence of the catalyst^24^. In the following, the catalyst is recycled and it enters the next cycle. The mechanism of this step is presented in detail in Fig. 10b.

FT-IR spectroscopy was accomplished for **3b** and **3c** derivatives to confirm the structure of *β*-ketoallylic methylsulfone products. The absorption bands of different functional groups of **3b** and **3c** are presented in Tables 5 and 6. The important characteristic absorption bands related to the sulfone group were observed in both **3b** and **3c** derivatives^25^.

**Table 5:** FT-IR data of **3b**.

Wavenumber (cm^−1^)	Assignments
3087	C–H stretching of terminal alkene
2942	C–H stretching of O-CH_3_
2926	C–H stretching of CH_2_
2843	C–H stretching of SO_2_Me
1649	Overlapping C=O, C=C stretching
1603	C=C stretching of aromatic
1511, 1462 and 1421	C–H bending of CH_3_ and CH_2_ aliphatic groups
1302 and 1109	SO_2_ asymmetric stretching

**Table 6:** FT-IR data of **3c**.

Wavenumber (cm^−1^)	Assignments
3113	C–H stretching of terminal alkene
3071	C–H stretching of aromatic
Less than 3000	C–H stretching of SMe and CH_2_
1700	C=O stretching
1666	C=C stretching
1412	C–H bending of CH_3_ and CH_2_
1521 and 1351	NO_2_ asymmetric stretching
1297 and 1124	SO_2_ asymmetric stretching

#### Recyclability test

The reusability of magnetic Ag–Cu MOF was performed under the optimized reaction conditions. The procedure is described as follow: the nanocomposite was collected by an external magnetic field after each run and then the whole of the nanocomposite was washed with methanol and water, dried in the oven and prepared for the next run. As a result, this nanocomposite indicated the recyclability to produce *β*-ketoallyl methylsulfone without losing activity for three successive runs (Table 7).

**Table 7:** The recyclability of magnetic Ag–Cu MOF in the standard reaction conditions.

Run	1	2	3
Yield	96	91	89

### Computational studies and results

**Figure 11:**
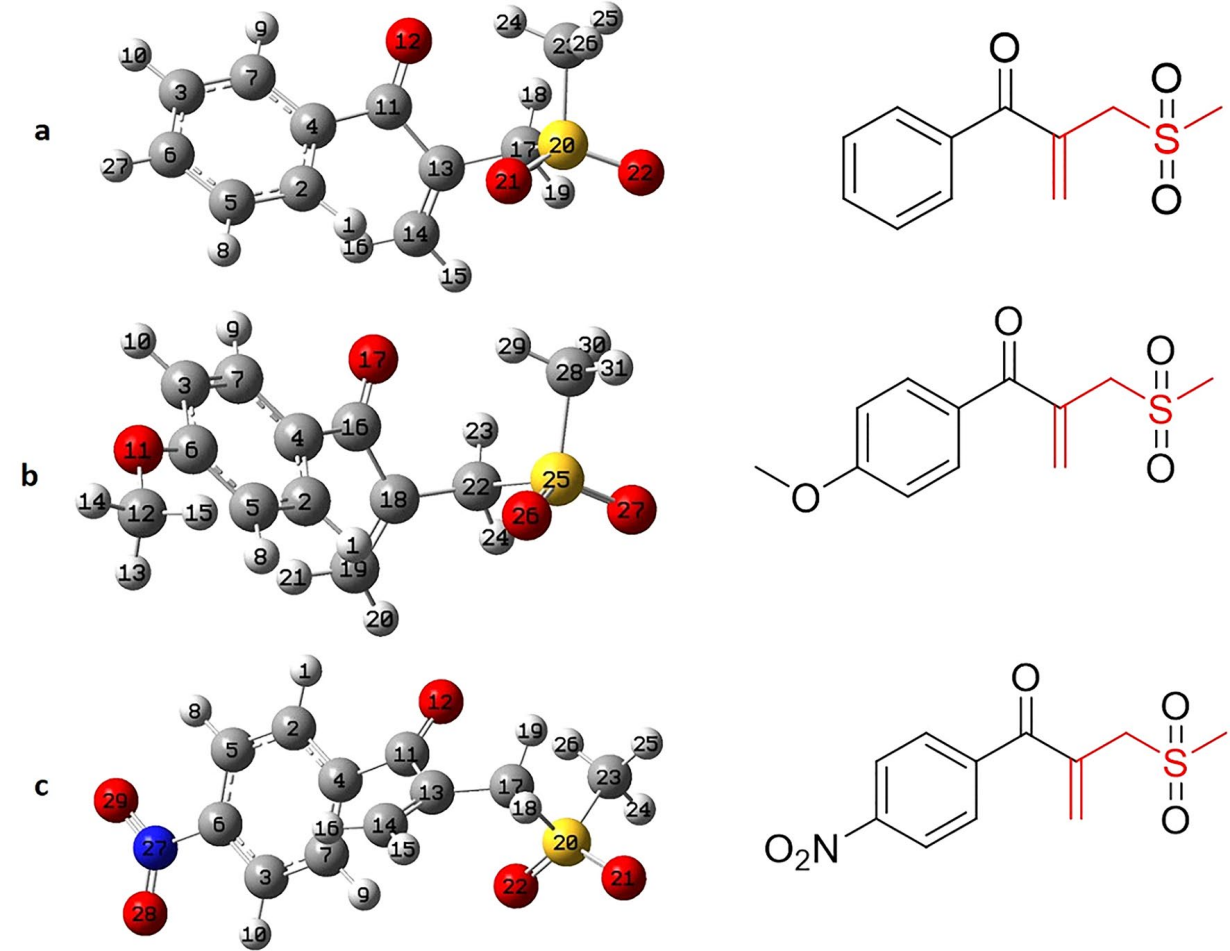
The B3LYP/6-311G + (d, p) optimized geometries of **3a**, **3b** and **3c**.

The electronic properties, Frontier molecular orbitals (FMO) analysis, IR and NMR spectra and other data of three type *β*-ketoallylic methylsulfone derivatives containing without substitution (**3a**), electron-rich (**3b**), and electron-deficient (**3c**) were investigated theoretically by utilizing Gaussian 09w software program^26^ with DFT/B3LYP method level using 6–311 + *G*(*d, p*) (Fig. 11).

#### Frontier molecular orbitals (FMOs)

**Figure 12:**
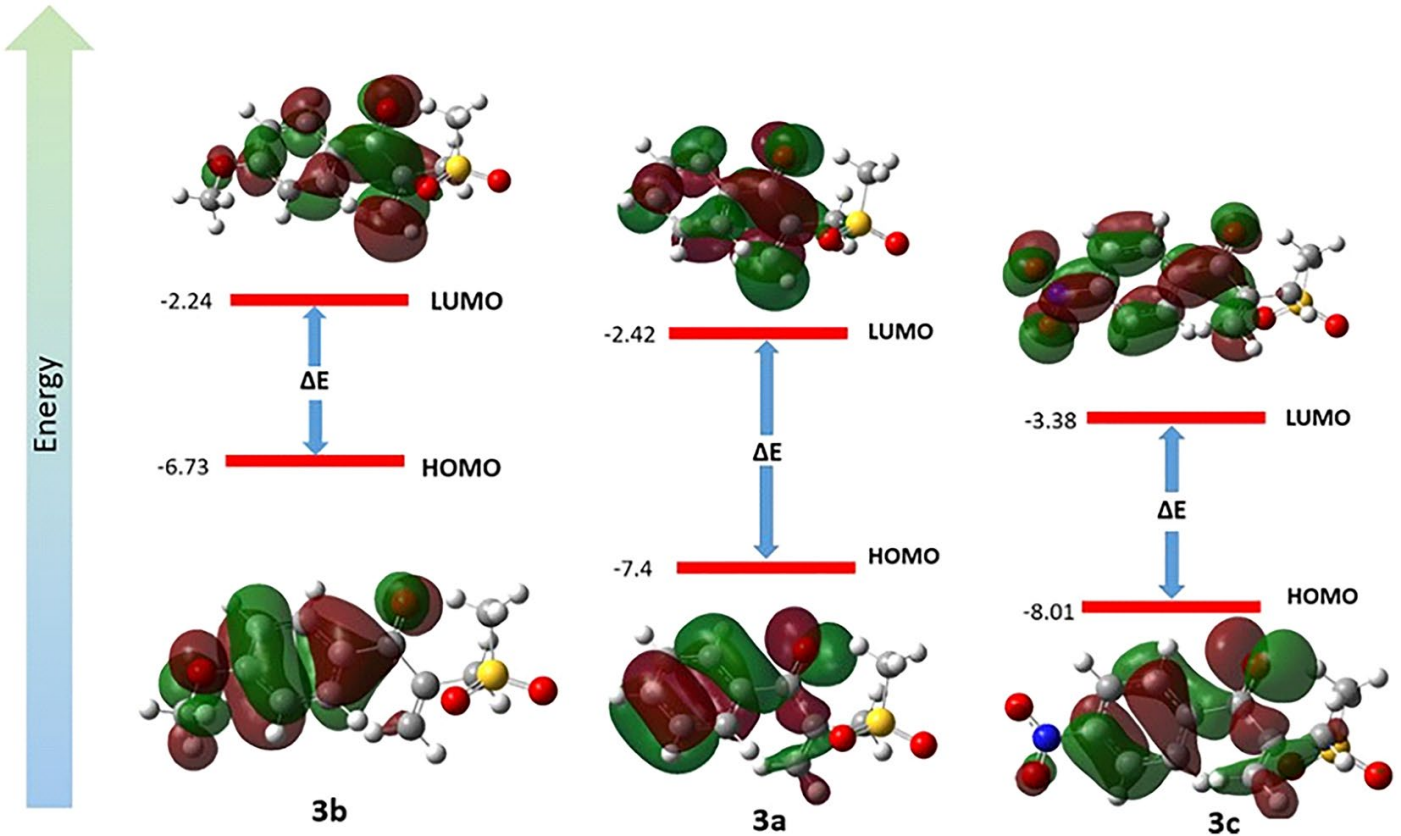
HOMO–LUMO energy level 3D-diagram of **3a**, **3b** and **3c** derivatives.

The gap energy (Eg) between the highest occupied molecular orbital (HOMO) and the lowest unoccupied molecular orbital (LUMO) of an organic molecule is a critical parameter that could predict the reactivity and stability of the molecule. There is an inverse relationship between the gap energy and reactivity^27^. Generally speaking, molecules with more gap energy and consequently more chemical hardness are less reactive^28^. The HOMO–LUMO structures with the energy level diagrams of **3a**, **3b** and **3c** are depicted in Fig. 12. Electronic properties of **3a**, **3b** and **3c** such as HOMO–LUMO energy gap (Eg), ionization potential (I), chemical softness (S), electron affinity (A), global electronegativity (χ), chemical hardness (η), chemical potential (μ), chemical softness (S), global electro-philicity (ω) were calculated based on the literature^29^. As reported in Table S1, the derivative **3a** has the highest energetic gap and the product **3b** has the lowest Eg making it the softest molecule. The product **3b** is the best electron donor due to having the highest of HOMO energy level. Furthermore, the product **3c** as an electron-deficient derivative has the lowest HOMO and LUMO energy levels and it can be the best electron acceptor. The two quantum chemical descriptors such as I and A are so applicable for prediction reactivity and calculation χ and η and related to the single electron orbital energies of the HOMO and LUMO, respectively. The product **3b** with the lowest potential ionization is the better electron donor while **3c** has the largest amount of electron affinity and it is better electron acceptor. The χ amount for **3c** is the largest, thus it is more electron acceptor and also **3c** has the largest value of ω suggesting more electrophilicity. Remarkably, **3b** was predicted a soft derivative among of three products because of having the lowest energetic gap, hardness and the largest softness^30^.

#### Calculated NMR section

Optimized structures of **3b** and **3c** by B3LYP/6–31 + *G*(*d*, *p*) level were used to calculate the NMR in the chloroform-*d*_*6*_ as solvent (the solvent for experimental NMR was chloroform-*d*_*6*_) with GIAO method, then we employed calculated data from output files. It was computed chemical shifts of hydrogens and carbons of **3b** and **3c** based on the articles^31^, and formula^32^ (Table S2) and the results are shown in Tables S3 and S4. The linear relationship was observed between experimentally data and calculated data of ^1^H-NMR and ^13^C-NMR of **3b** (Figure S1) and **3c** (Figure S2) with R^2^ ≈ 1, then there is a good agreement between experimental and calculated NMR of **3b** and **3c** products.

#### Calculated IR section

Based on the pervious article^33^, we obtained calculated IR of **3b** and **3c** by B3LYP/6–311 + *G*(*d, p*) basis set which results displayed good agreement with the experimental IR data for the both derivatives (Figures S3 and S4).

#### Electrostatic potential map

**Figure 13:**
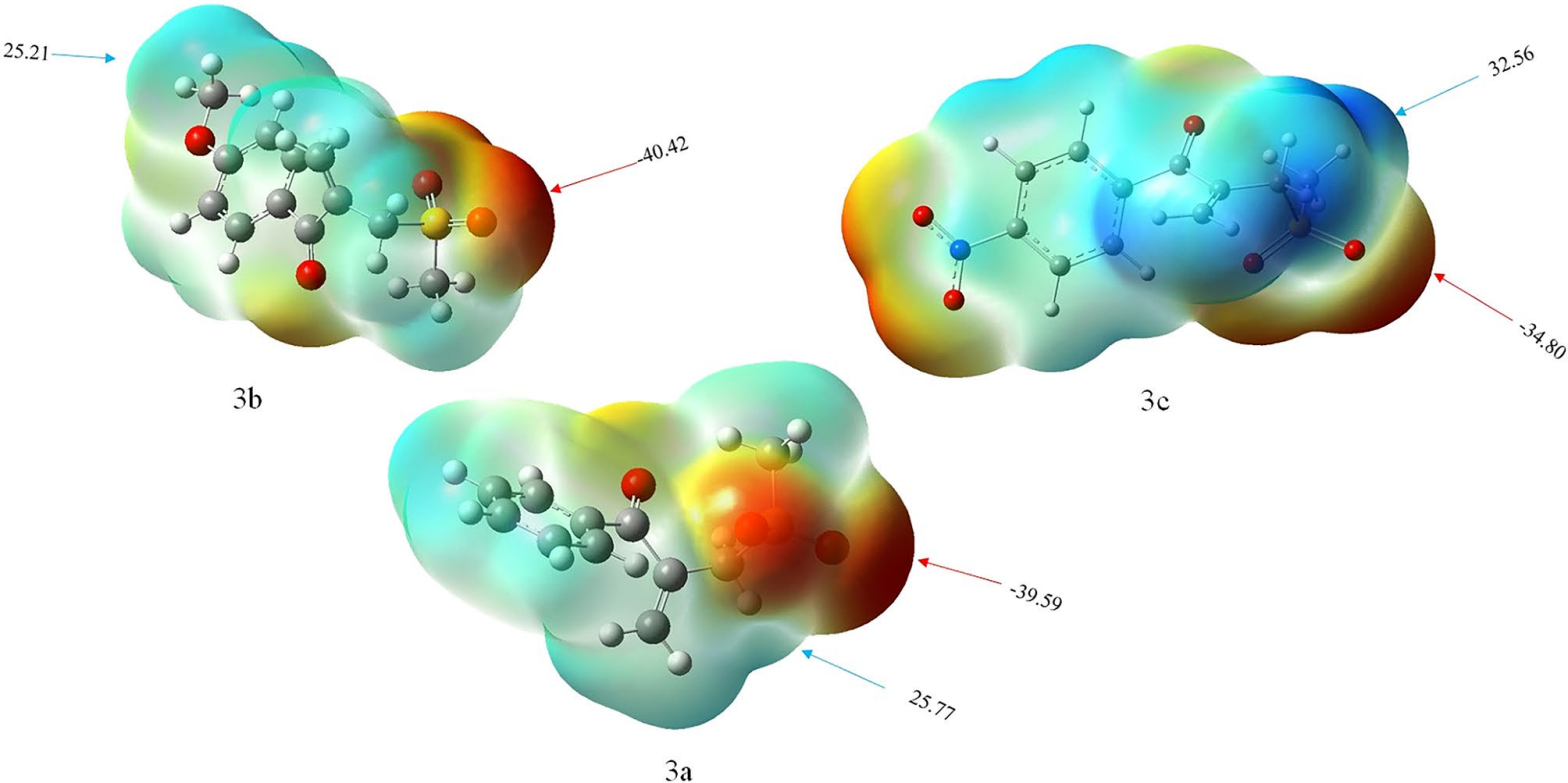
The EPS of **3a**, **3b** and **3c**.

The electrostatic potential provides a simple way to predict the chemical reactivity and effects of the different geometries on interacts^34^. To look for nucleophilic and electrophilic centers of **3a**, **3b** and **3c**, molecular electrostatic potential (ESP) analysis was performed after optimization the structures. The blue and red colors of the electrostatic potential map indicate the lack of electron and extra electron, respectively^35^. The results of ESP analysis of **3a**, **3b** and **3c** with the energy maximum and minimum points (kcal/mol) are presented in Fig. 13. As can be seen in this figure, the concentration of blue color is on the benzene, alkyl and alkenyl groups while the concentration of red color is on oxygen atoms.

## Conclusion

In conclusion, we have developed β-ketoallylic methylsulfones one-pot synthesis by DMSO and acetophenones. Some prominent features of this work are new and wide functional group tolerance, high atom economy, and recoverable bimetallic catalyst allowing to access the various *β*-ketoallylic methylsulfone derivatives with 74–96% yields. In addition, a detailed mechanistic pathway has been presented for a better understanding the role of materials in proceeding with the reaction. Important to note that a direct C(*sp*^3^)–H bond activation and functionalization of versatile acetophenones occurred for the first time through the magnetic bimetallic Ag–Cu MOF catalyst. Furthermore, the present work offers a synthetic protocol to generate a new chromane-4-one derivative with the intramolecular Michael-addition of the related *β*-ketoallyl methylsulfone product. Finally, computational studies have been done to investigate the electronic properties, reactivity, and stability of some obtained products.

### Supplementary Information

Supplementary Information.

## Data Availability

The datasets used and/or analysed during the current study available from the corresponding author on reasonable request.

## References

[1] Chen S, Li Y, Wang M, Jiang X (2020). General sulfone construction via sulfur dioxide surrogate control. Green Chem..

[2] Kumar N, Kumar A (2019). Amino acid-catalyzed direct synthesis of β-keto sulfones via aerobic difunctionalization of terminal alkynes in an aqueous medium. ACS Sustain. Chem. Eng..

[3] Nambo M, Maekawa Y, Crudden CM (2022). Desulfonylative transformations of sulfones by transition-metal catalysis, photocatalysis, and organocatalysis. ACS Catal..

[4] Nambo M, Keske EC, Rygus JP, Yim JC-H, Crudden CM (2017). Development of versatile sulfone electrophiles for Suzuki–Miyaura cross-coupling reactions. ACS Catal..

[5] Cui H-L (2022). Recent advances in DMSO-based direct synthesis of heterocycles. Molecules.

[6] Patel OP, Anand D, Maurya RK, Yadav PP (2016). H_2_O_2_/DMSO-promoted regioselective synthesis of 3,3′-bisimidazopyridinylmethanes via intermolecular oxidative C (sp^2^)–H bond activation of imidazoheterocycles. J. Org. Chem..

[7] Jadhav SD, Singh A (2017). Oxidative annulations involving DMSO and formamide: K_2_S_2_O_8_ mediated syntheses of quinolines and pyrimidines. Org. Lett..

[8] Gao X (2015). NH_4_I-mediated three-component coupling reaction: Metal-free synthesis of β-alkoxy methyl sulfides from DMSO, alcohols, and styrenes. Org. Lett..

[9] Xie C (2017). Dimethyl sulfoxide involved one-pot synthesis of quinoxaline derivatives. J. Org. Chem..

[10] Liu Y-F, Ji P-Y, Xu J-W, Hu Y-Q, Liu Q, Luo W-P, Guo C-C (2017). Transition metal-free α-Csp^3^-H methylenation of ketones to Form C=C bond using dimethyl sulfoxide as carbon source. J. Org. Chem..

[11] Liu Y, Hu Y, Cao Z, Zhan X, Luo W, Liu Q, Guo C (2018). Copper-catalyzed aerobic oxidative cyclization of anilines, aryl methyl ketones and DMSO: Efficient assembly of 2-arylquinolines. Adv. Synth. Catal..

[12] Liu Y (2019). Direct assembly of polysubstituted furans via C(sp^3^)−H bond functionalization by using dimethyl sulfoxide as a dual synthon. Adv. Synth. Catal..

[13] Xu X (2019). Cobalt(III)-catalyzed and dimethyl sulfoxide-involved cross-coupling of ketones and amides for direct synthesis of β-amino ketones. Adv. Synth. Catal..

[14] Kalari S, Karale UB, Rode HB (2022). Selectfluor-mediated synthesis of β-acyl allyl sulfones/β-acyl allyl benzotriazoles from ketones/acetylenes, aryl sulfinates/benzotriazole, and DMSO as a dual-carbon synthon. J. Org. Chem..

[15] Xiao F, Liu C, Wang D, Huang H, Deng G-J (2018). Concise synthesis of ketoallyl sulfones through an iron-catalyzed sequential four-component assembly. Green Chem..

[16] Li H, Li B-J, Shi Z-J (2011). Challenge and progress: Palladium-catalyzed sp^3^ C–H activation. Catal. Sci. Technol..

[17] Moghaddam FM, Jarahiyan A, Heidarian Haris M, Pourjavadi A (2021). An advancement in the synthesis of nano Pd@magnetic amine-functionalized UiO-66-NH_2_ catalyst for cyanation and O-arylation reactions. Sci. Rep..

[18] El-Yazeed WA, Ahmed AI (2019). Monometallic and bimetallic Cu–Ag MOF/MCM-41 composites: Structural characterization and catalytic activity. RSC Adv..

[19] Sun C-Y (2009). Highly stable crystalline catalysts based on a microporous metal–organic framework and polyoxometalates. J. Am. Chem. Soc..

[20] Ma R, Yang P, Ma Y, Bian F (2018). Facile synthesis of magnetic hierarchical core–shell structured Fe_3_O_4_@PDA-Pd@MOF nanocomposites: Highly integrated multifunctional catalysts. ChemCatChem.

[21] Gupta NK, Bae J, Kim KS (2021). Bimetallic Ag–Cu-trimesate metal–organic framework for hydrogen sulfide removal. New J. Chem..

[22] Gupta NK, Kim S, Bae J, Kim KS (2021). Chemisorption of hydrogen sulfide over copper-based metal–organic frameworks: Methanol and UV-assisted regeneration. RSC Adv..

[23] Liu Y (2017). Transition metal-free C(sp^3^)–H bond coupling among three methyl groups. Chem. Commun..

[24] Kupwade R, Khot S, Lad U, Desai U, Wadgaonkar P (2017). Catalyst-free oxidation of sulfides to sulfoxides and diethylamine catalyzed oxidation of sulfides to sulfones using Oxone as an oxidant. Res. Chem. Intermed..

[25] Chiu Y-C, Chou I-C, Tseng W-C, Ma C-CM (2008). Preparation and thermal properties of diglycidylether sulfone epoxy. Polym. Degrad. Stab..

[26] Frisch, M. J. *Gaussian 09 revision A*. 02. (2009).

[27] Gocen T, Bayarı SH, Guven MH (2017). Linoleic acid and its potassium and sodium salts: A combined experimental and theoretical study. J. Mol. Struct..

[28] Parr RG, Szentpály LV, Liu S (1999). Electrophilicity index. J. Am. Chem. Soc..

[29] Parr RG, Yang W (1984). Density functional approach to the frontier-electron theory of chemical reactivity. J. Am. Chem. Soc..

[30] Fleming I (2011). Molecular Orbitals and Organic Chemical Reactions.

[31] Subramanian N, Sundaraganesan N, Jayabharathi J (2010). Molecular structure, spectroscopic (FT-IR, FT-Raman, NMR, UV) studies and first-order molecular hyperpolarizabilities of 1, 2-bis (3-methoxy-4-hydroxybenzylidene) hydrazine by density functional method. Spectrochim. Acta A Mol. Biomol..

[32] http://cheshirenmr.info.

[33] Bayrakdar A, Mert S, Kasımoğulları R, Bangaru S, Manivannan P (2022). Synthesis, spectroscopic (FT-IR and NMR), DFT and molecular docking studies of ethyl 1-(3-nitrophenyl)-5-phenyl-3-((4-(trifluoromethyl) phenyl) carbamoyl)-1H-pyrazole-4-carboxylate. Res. Chem. Intermed..

[34] Domingo LR, Ríos-Gutiérrez M, Pérez P (2016). Applications of the conceptual density functional theory indices to organic chemistry reactivity. Molecules.

[35] Pearson RG (1992). The electronic chemical potential and chemical hardness. J. Mol. Struct..

