# Supplementary material for: β-Ketoallylic methylsulfones synthesis via inert C(sp3)–H bond activation by magnetic Ag–Cu MOF
Source: Sci Rep. 2023 Dec 18;13:22518. doi: 10.1038/s41598-023-49670-5 (PMC10728156; doi:10.1038/s41598-023-49670-5)
Supplement: Supplementary file 1 — Supplementary Information. [file 41598_2023_49670_MOESM1_ESM.pdf]

# Supporting Information

# $\beta$-ketoallylic methylsulfones synthesis via inert C(sp$^3$)-H bond activation by magnetic Ag-Cu MOF

**Firouz Matloubi Moghaddam *, Atefeh Jarahiyan, Parisa Yaqubnezhad Pazoki**

*Laboratory of Organic Synthesis and Natural Products, Department of Chemistry, Sharif University of Technology, Tehran, Iran*

*Correspondence*

*Firouz Matloubi Moghaddam, Laboratory of Organic Synthesis and Natural Products, Department of Chemistry, Sharif University of Technology, Azadi Street, PO Box 111559516, Tehran, Iran. Email: matloubi@sharif.edu*

## Contents:

NMR data of synthesized compounds....................S3-S7

Table S1 .................... S8

Table S2 .................... S8

Table S3 .................... S9

Table S4 .................... S10

Figure S1 .................... S11

Figure S2 .................... S12

Figure S3 .................... S13

Figure S4 .................... S14

## NMR data of the products

**2-((methylsulfonyl)methyl)-1-phenylprop-2-en-1-one**

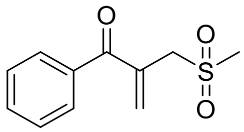


Yellow Oil. $^{1}$**H NMR** (500 MHz, $CDCl_3$): δ 7.79 (d, J = 7.1 Hz, 2H), 7.60 (t, J = 7.3 Hz, 1H), 7.48 (t, J = 7.5 Hz, 2H), 6.46 (s, 1H), 6.16 (s, 1H), 4.24 (s, 2H), 2.95 (s, 3H); $^{13}$**C NMR** (125 MHz, $CDCl_3$) δ 195.45, 136.12, 135.69, 132.91, 129.67, 129.39, 128.35, 56.62, 41.09.

**1-(4-methoxyphenyl)-2-((methylsulfonyl)methyl)prop-2-en-1-one**

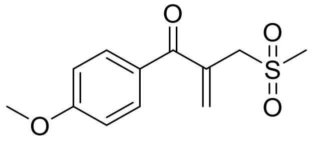


Yellow Solid. $^{1}$**H NMR** (500 MHz, $CDCl_3$): δ 7.83 (d, J = 8.9 Hz, 2H), 6.96 (d, J = 8.8 Hz, 2H), 6.36 (s, 1H), 6.09 (s, 1H), 4.20 (s, 2H), 3.88 (s, 3H), 2.93 (s, 3H); $^{13}$**C NMR** (125 MHz, $CDCl_3$) δ 194.04, 163.57, 135.59, 132.32, 131.94, 128.53, 113.79, 113.59, 57.07, 48.36, 40.98.

**2-((methylsulfonyl)methyl)-1-(4-nitrophenyl)prop-2-en-1-one**

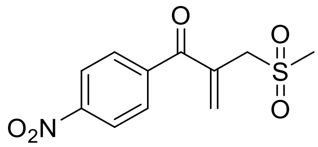


Yellow Solid. $^{1}$**H NMR** (500 MHz, $CDCl_3$): δ 8.35 (d, J = 8.8 Hz, 2H), 7.96 (d, J = 8.8 Hz, 2H ), 6.55 (s, 1H), 6.14 (s, 1H), 4.30 (s, 2H), 3.02 (s, 3H); $^{13}$**C NMR** (125 MHz, $CDCl_3$) δ 194.03, 150.80, 135.63, 131.21, 130.60, 123.77, 123.63, 100.04, 56.56, 41.67.

**1-(4-bromophenyl)-2-((methylsulfonyl)methyl)prop-2-en-1-one**

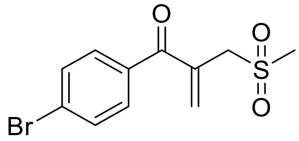


Yellow Solid. $^{1}$**H NMR** (500 MHz, $CDCl_3$): δ 7.69 (d, J = 8.4 Hz, 2H), 7.65 (d, J = 6.8 Hz, 2H), 6.46 (s, 1H), 6.13 (s, 1H), 4.25 (s, 2H), 2.98 (s, 3H); $^{13}$**C NMR** (125 MHz, $CDCl_3$) δ 194.10, 135.24, 134.53, 131.44, 130.76, 129.17, 127.74, 56.37, 40.94.

**1-(2-chlorophenyl)-2-((methylsulfonyl)methyl)prop-2-en-1-one**

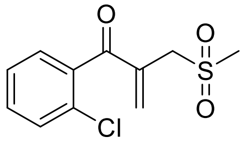

Yellow Solid. **$^1$H NMR** (500 MHz, $CDCl_3$): δ 7.60 (d, J = 7.5 Hz, 1H), 7.41-7.34 (m, 3H), 6.65 (s, 1H), 6.16 (s, 1H), 4.24 (s, 2H), 3.00 (s, 3H); **$^{13}$C NMR** (125 MHz, $CDCl_3$) δ 194.86, 139.08, 136.89, 136.70, 132.84, 131.75, 129.11, 127.35, 54.71, 41.31.

**1-(4-fluorophenyl)-2-((methylsulfonyl)methyl)prop-2-en-1-one**

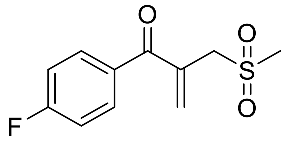

Yellow Solid. **$^1$H NMR** (500 MHz, $CDCl_3$): δ 7.87 (dd, J = 8.7, 5.4 Hz, 2H), 7.20 (t, J = 7.3 Hz, 2H),6.44 (s, 1H), 6.13 (s, 1H), 4.25 (s, 2H), 2.98 (s, 3H).

**2-((methylsulfonyl)methyl)-1-(thiophen-2-yl)prop-2-en-1-one**

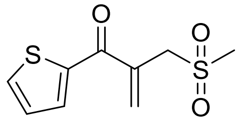

Yellow Solid. **$^1$H NMR** (500 MHz, $CDCl_3$): δ 7.80 – 7.76 (m, 2H), 7.22 – 7.18 (m, 1H), 6.41 (s, 2H), 4.19 (s, 2H), 2.96 (s, 3H).

**2-((methylsulfonyl)methyl)-1-(pyridin-3-yl)prop-2-en-1-one**

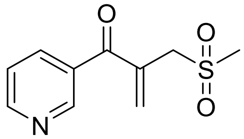

Yellow Solid. **$^1$H NMR** (500 MHz, $CDCl_3$): 9.04 (s, 1H), 8.86 (d, J = 7.7 Hz, 1H), 8.15 (d, J = 7.8 Hz, 1H), 7.55 – 7.42 (m, 1H), 6.55 (s, 1H), 6.20 (s, 1H), 4.28 (s, 2H), 3.02 (s, 3H).

**2-((methylsulfonyl)methyl)-1-(naphthalen-1-yl)prop-2-en-1-one**

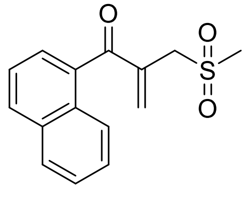

Yellow Solid. **$^1$H NMR** (500 MHz, $CDCl_3$): 8.78 (d, J = 8.7 Hz, 1H), 8.03 (d, J = 8.2 Hz, 1H), 7.96 (t, J = 10.3 Hz, 1H), 7.91 (d, J = 8.2 Hz, 1H), 7.64 (t, J = 7.7 Hz, 1H), 7.57 (t, J = 7.5 Hz, 1H), 7.53 (t, J = 7.7 Hz, 1H), 5.96 (s, 1H), 5.68 (s, 1H), 4.20 (s, 2H), 2.99 (s, 3H).

**1-(4-chlorophenyl)-2-((methylsulfonyl)methyl)prop-2-en-1-one**

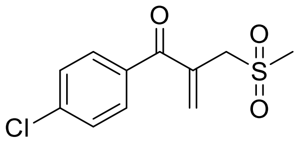

Yellow Solid. **$^1$H NMR** (500 MHz, $CDCl_3$): δ 7.90 (d, J = 8.4 Hz, 2H), 7.44 (d, J = 8.4 Hz, 2H), 6.44 (s, 1H), 6.14 (s, 1H), 4.21 (s, 2H), 2.97 (s, 3H).

**1-([1,1'-biphenyl]-4-yl)-2-((methylsulfonyl)methyl)prop-2-en-1-one**

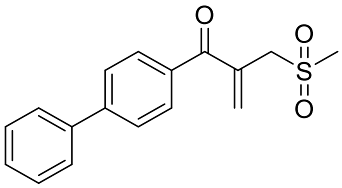

Yellow Solid. **$^1$H NMR** (500 MHz, $CDCl_3$): δ 8.04 (d, J = 7.7 Hz, 2H), 7.69 (d, J = 7.6 Hz, 2H), 7.63 (d, J = 7.8, Hz, 2H), 7.47 (t, J = 7.3 Hz, 2H), 7.40 (t, J = 7.1 Hz, 1H), 5.98 (s, 1H), 5.72 (s, 1H), 4.20 (s, 2H), 2.98 (s, 3H).

**2-((methylsulfonyl)methyl)-1-(p-tolyl)prop-2-en-1-one**

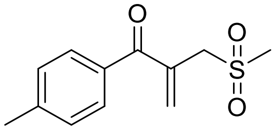

Yellow Solid. **$^1$H NMR** (500 MHz, $CDCl_3$): δ 7.88 (d, J = 7.5 Hz, 1H), 7.28 (d, J = 7.6 Hz, 1H), 6.46 (s, 1H), 6.13 (s, 1H), 4.21 (s, 2H), 2.96 (s, 3H), 2.43 (s, 3H).

**1-(4-ethoxyphenyl)-2-((methylsulfonyl)methyl)prop-2-en-1-one**

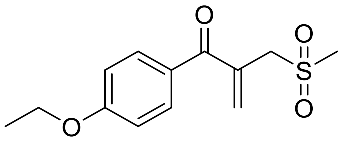

Yellow Solid. **$^1$H NMR** (500 MHz, $CDCl_3$): δ 7.92 (d, J = 8.2 Hz, 2H), 6.91 (d, J = 8.8 Hz, 2H), 6.34 (s, 1H), 6.10 (s, 1H), 4.19 (s, 2H), 4.09 (q, 2H), 2.94 (s, 3H), 1.44 (t, J = 7.0 Hz, 3H).

**2-((methylsulfonyl)methyl)-1-(3-nitrophenyl)prop-2-en-1-one**

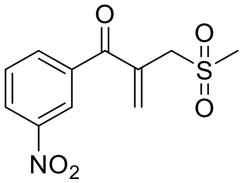

Yellow Solid. **$^1$H NMR** (500 MHz, $CDCl_3$): δ 8.80 (s, 1H), 8.45 (d, J = 7.2 Hz, 1H), 8.32 (d, J = 7.7 Hz, 1H), 7.72 (t, J = 7.9 Hz, 1H), 6.54 (s, 1H), 6.15 (s, 1H), 4.28 (s, 2H), 3.02 (s, 3H).

**2-(2-((methylsulfonyl)methyl)acryloyl)phenyl acetate**

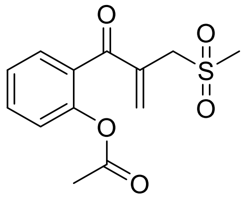

Yellow Solid. **$^{1}$H NMR** (500 MHz, $CDCl_3$): δ 7.84 (d, J = 7.8 Hz, 1H), 7.57 (t, J = 8.0 Hz, 1H), 7.35 ( t, J = 7.9 Hz, 1H), 7.14 (d, J = 8.1 Hz, 1H), 6.65 (s, 1H), 6.16 (s, 1H), 4.24 (s, 2H), 2.99 (s, 3H), 2.38 (s, 3H).

**4-(2-((methylsulfonyl)methyl)acryloyl)phenyl acetate**

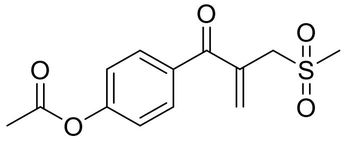

Yellow Solid. **$^{1}$H NMR** (500 MHz, $CDCl_3$): δ 8.02 (d, J = 6.9 Hz, 2H), 7.22 (d, J = 8.5 Hz, 2H), 6.44 (s, 1H), 6.13 (s, 1H), 4.22 (s, 2H), 2.98 (s, 3H), 2.35 (s, 3H).

**1-(4-hydroxyphenyl)-2-((methylsulfonyl)methyl)prop-2-en-1-one**

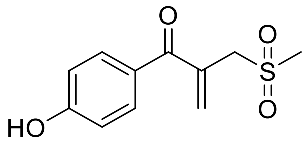

Yellow Solid. **$^{1}$H NMR** (500 MHz, $CDCl_3$): δ 9.98 (s, 1H), 7.77 (d, J = 8.3 Hz, 2H), 6.91 (t, J = 8.6 Hz, 2H), 6.36 (s, 1H), 6.10 (s, 1H), 4.23 (s, 2H), 2.93 (s, 3H).

**N-(4-(2-((methylsulfonyl)methyl)acryloyl)phenyl)acetamide**

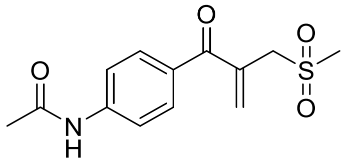

Yellow Solid. **$^{1}$H NMR** (500 MHz, $CDCl_3$): δ 7.95 (d, J = 6.6 Hz, 2H), 7.71 (s, 1H), 7.65 (d, J = 5.8 Hz, 2H), 6.43 (s, 1H), 6.15 (s, 1H), 4.24 (s, 2H), 2.92 (s, 3H), 2.24 (s, 3H).

**3-((methylsulfonyl)methyl)chroman-4-one**

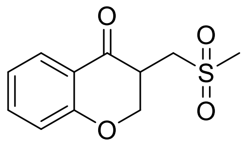

Yellow Solid. **$^{1}$H NMR** (500 MHz, $CDCl_3$): δ 7.92 (d, J = 7.7 Hz, 1H), 7.59 – 7.53 (m, 1H), 7.12 – 7.06 (m, 1H), 7.04 (d, J = 7.8 Hz, 1H), 4.94 (dd, J = 11.3, 5.4 Hz, 1H), 4.37 (t, J = 11.8 Hz, 1H), 3.92 (d, J = 14.9 Hz, 1H), 3.63-3.54 (m, 1H), 3.08 (s, 3H), 2.96 (dd, J = 14.5, 8.4 Hz, 1H); **$^{13}$C NMR** (125 MHz, $CDCl_3$) δ 194.15, 160.03, 136.77, 133.57, 127.68, 122.01, 118.14, 72.80, 54.81, 44.01, 41.71.

**1-(4-methoxyphenyl)-2-(((methyl-*d*$_3$)sulfonyl)methyl-*d*$_2$)prop-2-en-1-one-3,3-*d*$_2$**

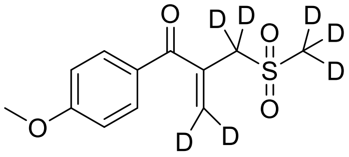

Yellow Oil. $^{1}$**H NMR** (500 MHz, $CDCl_3$): δ 7.99 (d, J = 7.7 Hz, 2H), 6.98 (d, J = 7.5 Hz, 2H), 3.90 (s, 3H).

**1-(4-methoxyphenyl)prop-2-en-1-one**

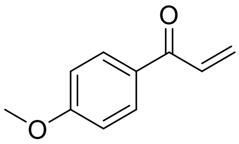

Yellow Oil. $^{1}$**H NMR** (500 MHz, $CDCl_3$): δ 7.93 (d, J = 7.5 Hz, 2H), 7.23 – 7.07 (m, 1H), 6.92 (d, J = 7.6 Hz, 2H), 6.39 (dd, J = 17.1, 1.8 Hz, 1H), 5.84 (dd, J = 10.5, 1.8 Hz, 1H), 3.83 (s, 3H); $^{13}$**C NMR** (125 MHz, $CDCl_3$) δ 196.77, 163.39, 131.99, 130.70, 130.00, 129.53, 113.79, 55.36.

| Table S1. Global chemical descriptors for 3a, 3b and 3c derivatives by B3LYP/6-311+*G*(*d*, *p*). | | | |
|---|---|---|---|
| Method | 6-31+G(d,p) | | |
| Molecular descriptors energy | 3b | 3a | 3c |
| ***E**_HOMO_**(eV)*** | -6.73 | -7.4 | -8.01 |
| ***E**_LUMO_**(eV)*** | -2.24 | -2.42 | -3.38 |
| $E_g=\Delta E_{HOMO-LUMO}(eV)$ | -4.49 | -4.98 | -4.63 |
| ***I(eV)*** | 6.73 | 7.4 | 8.01 |
| ***A (eV)*** | 2.24 | 2.42 | 3.38 |
| ***χ (eV)*** | 4.485 | 4.91 | 5.695 |
| ***(η)(eV)*** | 2.245 | 2.49 | 2.31 |
| ***μ (eV)*** | -4.485 | -4.91 | -5.69 |
| ***(S)[(eV)−1]*** | 0.225 | 0.2 | 0.215 |
| ***(ω)[(eV)−1]*** | 4.48 | 4.84 | 7 |

| Geometry (opt & freq) | NMR (nmr=method) | $^1H$ | 13C | $^1H$ | $^{13}C$ |
|---|---|---|---|---|---|
| (gas phase) | (gio,scrf) | slope:-1.0103 | slope:-1.0222 | RMSD:0.1517 | RMSD:2.2700 |
| B3LYP/6-31+G(d,p) | B3LYP/6-31G(d) | intercept:32.2306 | intercept:182.1337 | $R^2$:0.9968 | $R^2$:0.9986 |

Table S2. The values and formula to calculate chemical shift in $CDCl_3$ that were reported in (http://cheshirenmr.info/)

$$\delta = \frac{intercept - \sigma}{-slope}$$

| Atom | $\delta_{B3LYP}$ (ppm) | $\delta_{exp}$ (ppm) | $\delta_{exp}$-$\delta_{B3LYP}$ |
|---|---|---|---|
| H1 | 7.81 | 7.83 | 0.02 |
| H8 | 6.63 | 6.96 | 0.33 |
| H9 | 7.76 | 7.83 | 0.07 |
| H10 | 6.81 | 6.96 | 0.15 |
| H13 | 3.75 | 3.88 | 0.13 |
| H14 | 4.07 | 3.88 | -0.19 |
| H15 | 3.76 | 3.88 | 0.12 |
| H20 | 6.11 | 6.36 | 0.25 |
| H21 | 5.86 | 6.09 | 0.23 |
| H23 | 4.69 | 4.2 | -0.49 |
| H24 | 3.36 | 4.2 | 0.84 |
| H29 | 2.4 | 2.93 | 0.53 |
| H30 | 2.42 | 2.93 | 0.51 |
| H31 | 2.43 | 2.93 | 0.5 |
| C2 | 118.57 | 132 | 13.43 |
| C3 | 102.26 | 113.79 | 11.53 |
| C4 | 113.32 | 128.53 | 15.21 |
| C5 | 94.02 | 113.59 | 19.57 |
| C6 | 145.04 | 163.57 | 18.53 |
| C7 | 115.79 | 132 | 16.21 |
| C12 | 44.61 | 48.36 | 3.75 |
| C16 | 177.1 | 194.04 | 16.94 |
| C18 | 123.98 | 135.59 | 11.61 |
| C19 | 119.31 | 132.32 | 13.01 |
| C22 | 55.49 | 57.07 | 1.58 |
| C28 | 36.84 | 40.98 | 4.14 |

Table S3. $^{1}$H-NMR and $^{13}$C-NMR chemical shifts in chloroform-$d_6$ of **3b** product.

| Atom | $\delta_{B3LYP}$ (ppm) | $\delta_{exp}$ (ppm) | $\delta_{exp}$-$\delta_{B3LYP}$ |
|---|---|---|---|
| H1 | 7.84 | 7.96 | 0.12 |
| H8 | 8.28 | 8.35 | 0.07 |
| H9 | 7.98 | 7.96 | -0.02 |
| H10 | 8.27 | 8.35 | 0.08 |
| H15 | 6.29 | 6.55 | 0.26 |
| H16 | 5.92 | 6.14 | 0.22 |
| H18 | 3.5 | 4.3 | 0.8 |
| H19 | 4.78 | 4.3 | -0.48 |
| H24 | 2.53 | 3.02 | 0.49 |
| H25 | 2.56 | 3.02 | 0.46 |
| H26 | 2.64 | 3.02 | 0.38 |
| C2 | 115.53 | 123.63 | 8.1 |
| C3 | 108.46 | 100.04 | -8.42 |
| C4 | 126.99 | 135.63 | 8.64 |
| C5 | 109.07 | 100.04 | -9.03 |
| C6 | 133.81 | 150.7 | 16.89 |
| C7 | 116.51 | 123.77 | 7.26 |
| C11 | 179.56 | 194.03 | 14.47 |
| C13 | 124.22 | 131.21 | 6.99 |
| C14 | 121.64 | 130.6 | 8.96 |
| C17 | 55.61 | 56.56 | 0.95 |
| C23 | 37.51 | 41.67 | 4.16 |

Table S4. $^{1}$H-NMR and $^{13}$C-NMR chemical shifts in chloroform-$d_6$ of **3c** product.

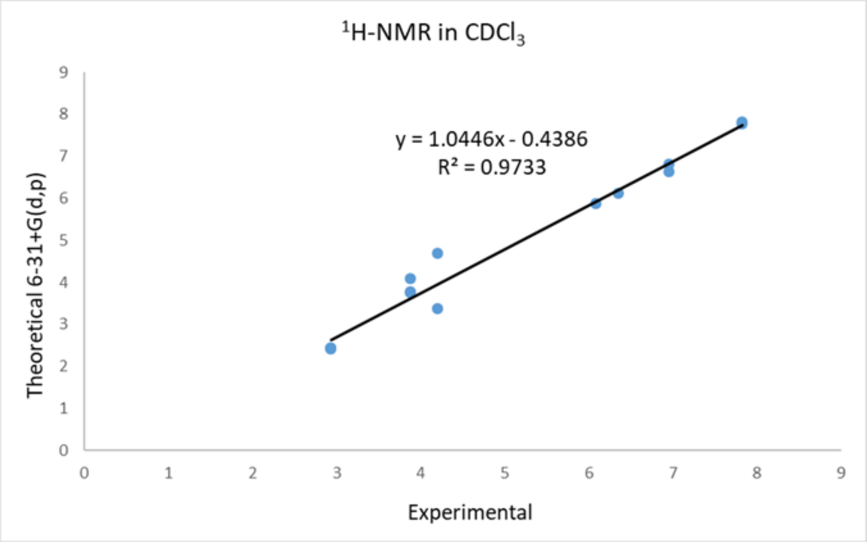


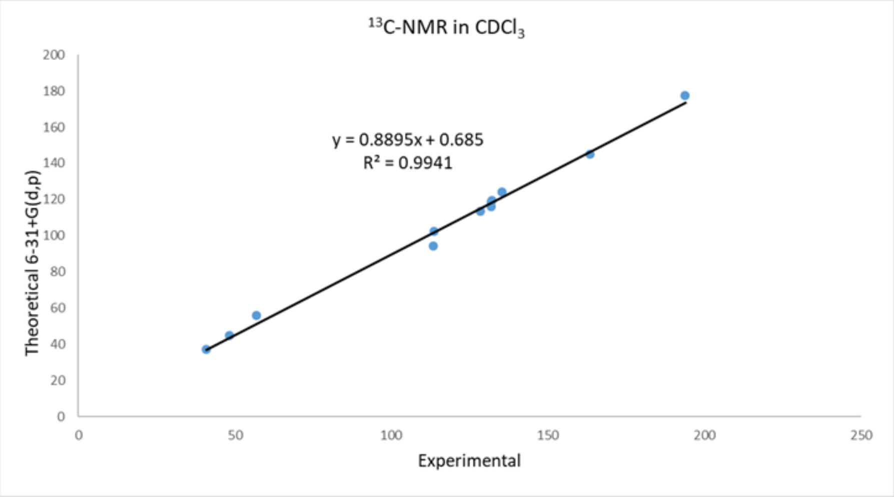


Figure S1. The linear correlation between experimental vs calculated $^{1}$H-NMR and $^{13}$C-NMR data of **3b**.

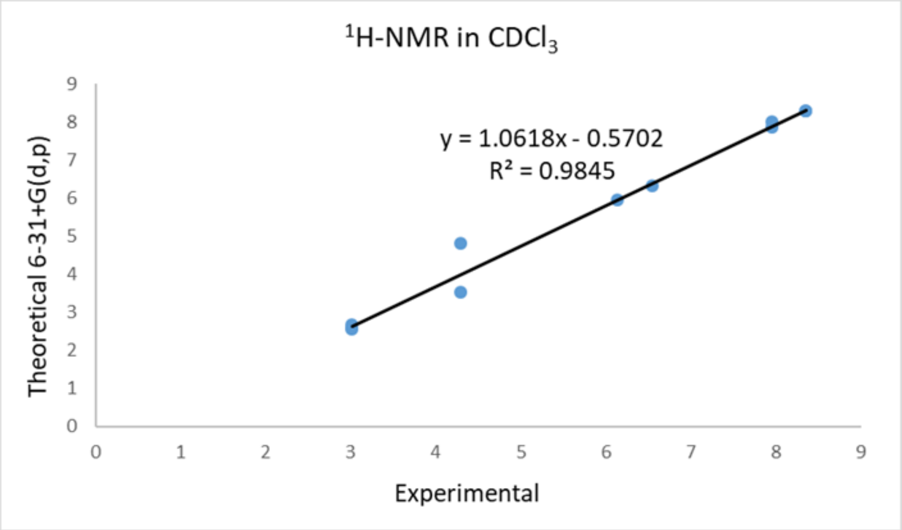


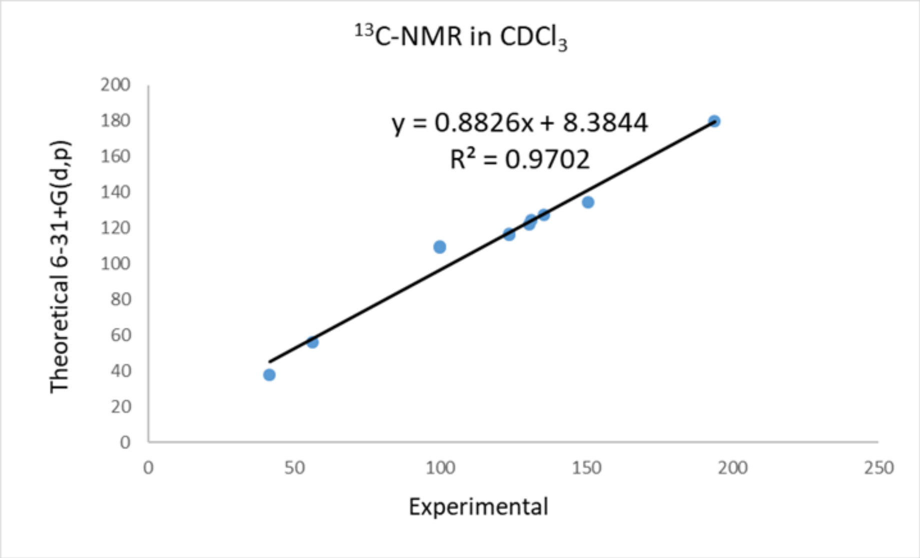


Figure S2. The linear correlation between experimental vs calculated $^{1}$H-NMR and $^{13}$C-NMR data of **3c**.

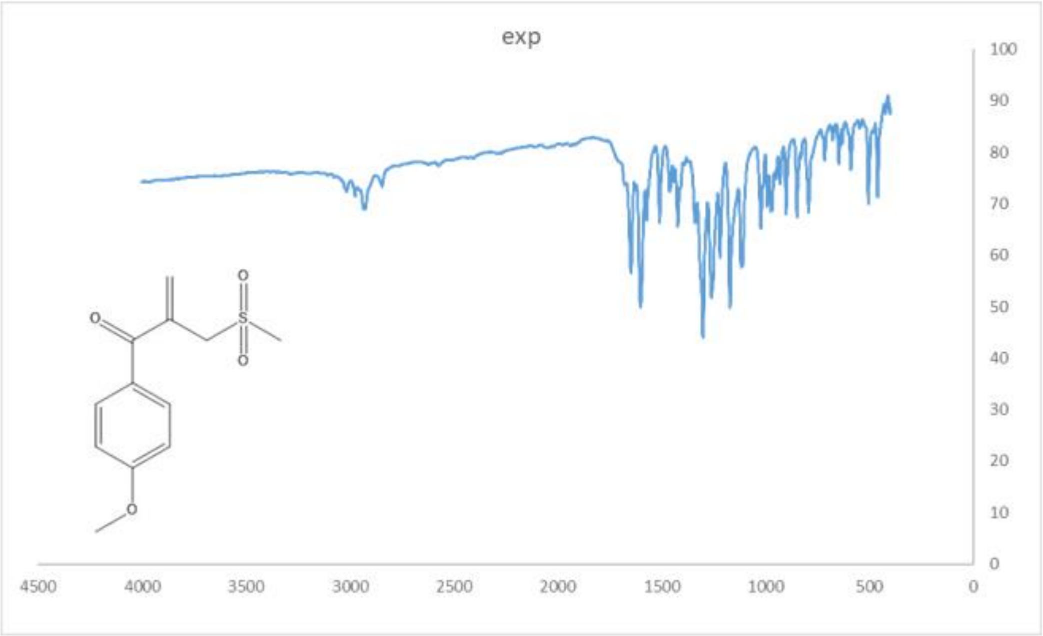


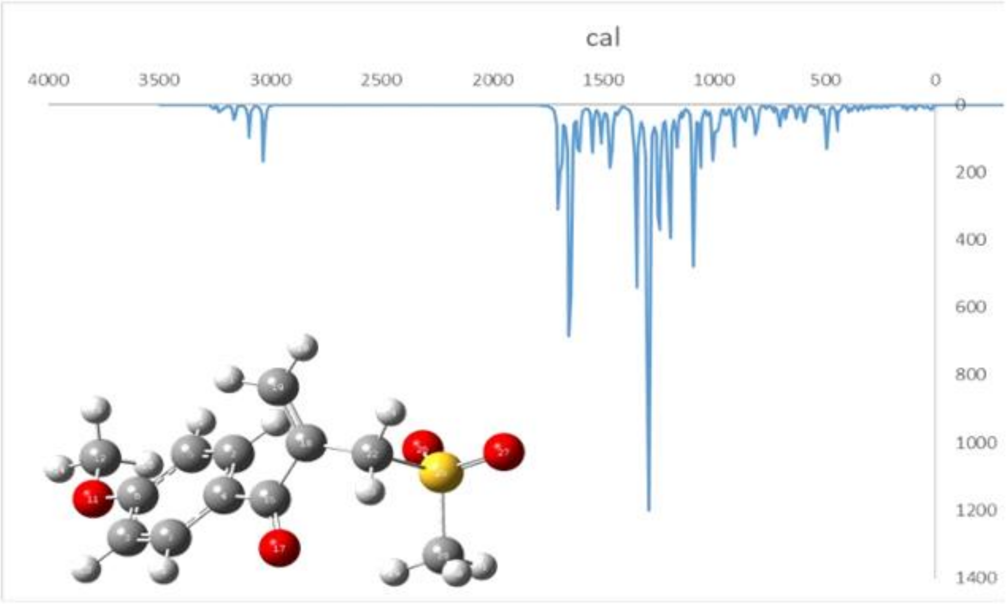


Figure S3. Experimental and theoretical B3LYP/6-311 + *G*(*d*, *p*) infrared spectra of **3b**.

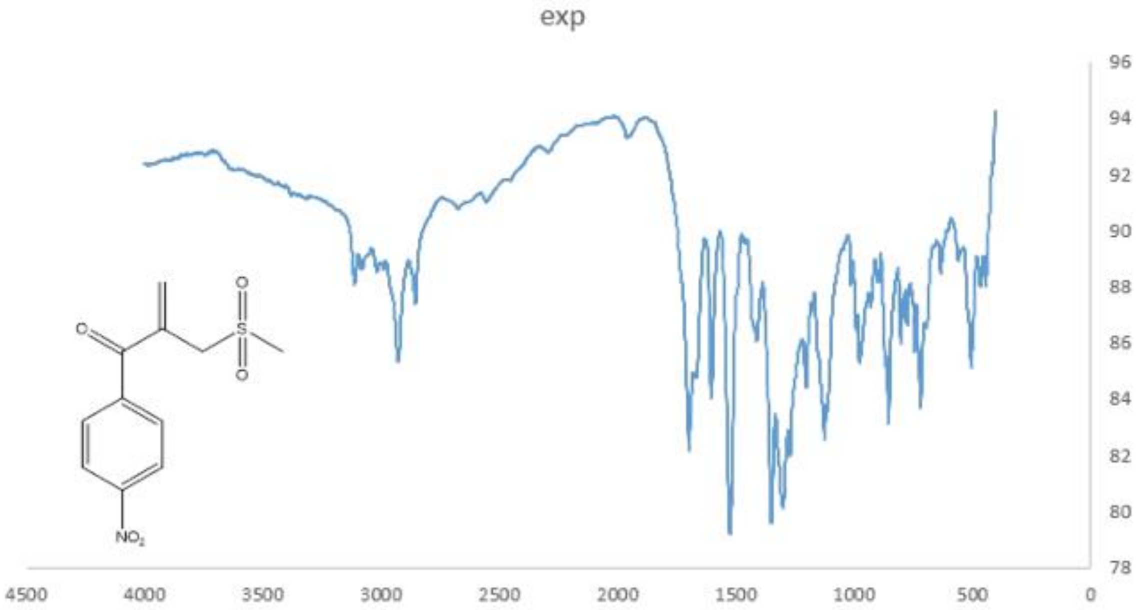


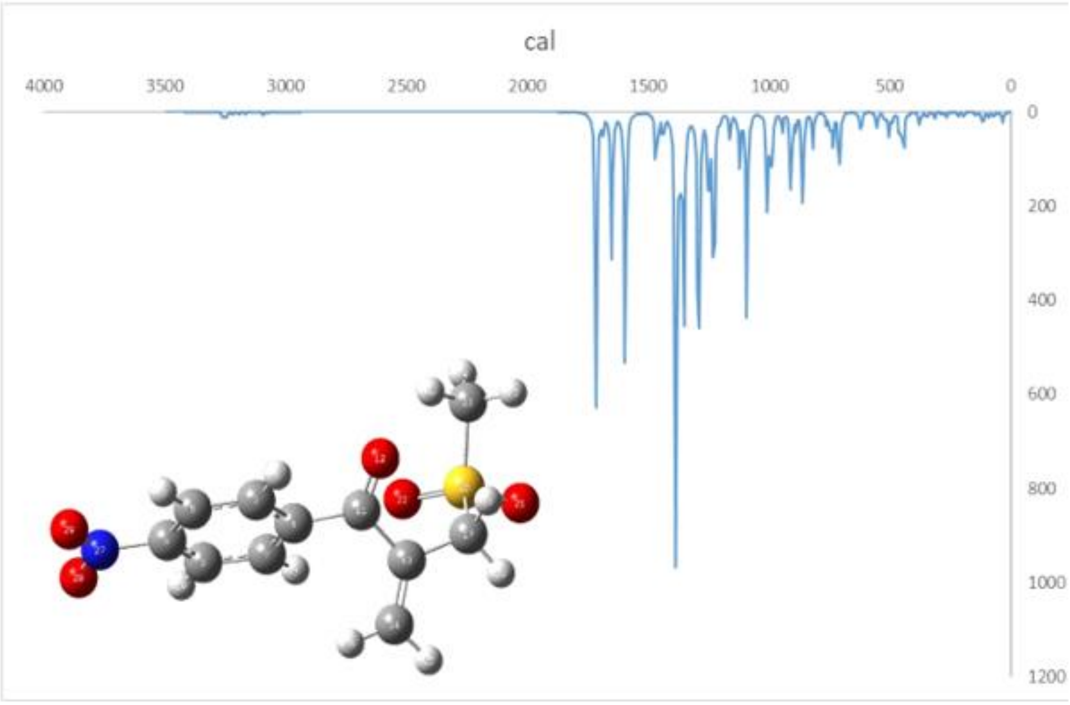


Figure S4. Experimental and theoretical B3LYP/6-311 + G(d, p) infrared spectra of **3c.**
